# The preventive effect of chlorogenic acid on cisplatin-induced acute kidney injury in mice

**DOI:** 10.3389/fvets.2026.1763548

**Published:** 2026-02-19

**Authors:** Zheng Hongya, Duan Yichang, Zhong Zheng, Zhu Yanzhu, Niu Wei, Huang Caoxing, Yin Baishuang

**Affiliations:** 1 College of Animal Science and Technology, Heilongjiang Bayi Agricultural University, Daqing, China; 2 Key Lab of Preventive Veterinary Medicine in Jilin Province, College of Animal Science and Technology, Jilin Agricultural Science and Technology University, Jilin, China; 3 College of Chemical Engineering, Nanjing Forestry University, Nanjing, China

**Keywords:** acute kidney injury, antioxidant capacity, chlorogenic acid, cisplatin, mice

## Abstract

Acute kidney injury (AKI) is a common clinical syndrome. Chlorogenic acid (CGA) is a natural polyphenol with antioxidant and anti-inflammatory properties. In this study, 60 male Kunming mice were randomly assigned to 6 groups: Control (CON), Cisplatin (CIS), CGA, CIS + CGA, CIS + furosemide (FUR), and FUR. Kidney injury markers, inflammatory indicators, antioxidant enzyme activities, oxidative products, antioxidant proteins, and kidney morphology were assessed using ELISA, histology, and Western blot. Preventive CGA supplementation significantly reduced levels of creatinine (Cr), BUN, KIM-1, and MDA, while restoring the enzymatic activities of SOD, GSH-Px, CAT, and T-AOC. CGA also increased the expression of Nrf2 and GCLC proteins and decreased the expression of Keap1 protein. Levels of IL-1β, IL-2, and IL-6 were reduced, while IL-10 levels were elevated. These results indicate that preventive CGA supplementation effectively mitigates CIS-induced AKI by enhancing antioxidant capacity, attenuating inflammatory responses, and ameliorating kidney structural damage.

## 1 Introduction

Acute kidney injury (AKI) is a common and potentially life-threatening condition characterized by a rapid increase in serum creatinine and a sudden reduction in urine output (1). The reported incidence of AKI in China increased from 8.5% in 2013 to 11.2% in 2017, with a mortality rate of 13.7% (2). Oliguria was the most common clinical symptom of AKI, and treatment generally focuses on promoting diuresis (3). Furosemide (FUR) is often used as a diuretic to prevent AKI, as it can dilate major blood vessels, reduce vascular resistance, and increase kidney cortical blood flow (4). However, prolonged FUR administration may lead to electrolyte disturbances and metabolic alkalosis (5).

The green peel of walnuts has antioxidant and anti-inflammatory activities (6). Chlorogenic acid (CGA), a polyphenolic secondary metabolite found in the green peel of walnuts. Exerts antioxidant, antibacterial, hepatoprotective, anti-inflammatory, and antiviral effects by scavenging free radicals (7, 8). Oxidative stress has emerged as the central pathophysiological mechanism mediating both the initiation and progression of AKI (9). Consequently, controlling oxidative stress represents a critical strategy for AKI. CGA has also been shown to attenuate kidney dysfunction in chronic kidney disease (10). In CKD, progressive kidney dysfunction results in the accumulation of indoxyl sulfate and hydroxy indoxyl sulfate. These toxins activated NADPH oxidase and disrupted the mitochondrial electron transport chain, leading to excessive ROS generation (11). However, it is not clear whether CGA in walnut green peel can improve AKI by inhibiting oxidative stress.

Therefore, in this study, AKI in mice was induced by cisplatin (CIS). The establishment of the AKI model was confirmed by measuring serum creatinine (Cr), blood urea nitrogen (BUN), kidney injury molecule-1 (KIM-1), and monitoring changes in the body weight of the mice. The effect of CGA from walnut green peel on AKI was evaluated by antioxidant activity, inflammatory markers, and kidney tissue analysis.

## 2 Materials and methods

### 2.1 Ethics statement

All experimental procedures were reviewed and approved by the Institutional Animal Care and Use Committee (IACUC) of the Jilin Agriculture Science and Technology University on 1 June 2025 and were conducted in accordance with its guidelines. The approved protocol number was LLSC202502007.

### 2.2 Establishment of animal models

Sixty male Kunming mice (7 weeks old, 35–40 g) were acclimated for 7 days and randomly assigned to six groups (n = 10 per group): Control (CON), CIS (CIS), Chlorogenic acid (CGA), CIS + CGA (CGA + CIS), CIS + Furosemide (CIS + FUR), and FUR (FUR). All animals had *ad libitum* access to food and water. The preventive CGA supplementation was conducted in the mice of the CGA group and the CIS + CGA group via oral gavage at a daily dose of 200 mg/kg, starting 14 days before CIS injection for preventive purposes. The dose of preventive CGA supplementation is consistent with the administered dosage of CGA reported in Feng’s experimental protocol (12). On the 11th day, a single intraperitoneal injection of CIS (20 mg/kg) was administered to mice in the CIS, CIS + CGA, and CIS + FUR groups. After a 12-h interval, mice in both the CIS + FUR and FUR groups were administered FUR via intraperitoneal injection at a dosage of 1 mg/kg per day for 3 consecutive days. This dosage is sufficient to activate renal tubular function without directly inducing renal damage associated with the drug’s intrinsic toxicity (13). The primary rationale for selecting a dose of 1 mg/kg of FUR for this experiment lies in its capacity to stimulate distal convoluted tubule secretion.

### 2.3 Evaluation of kidney injury models

Creatinine (Cr), BUN, and KIM-1 were quantified with commercial kits (Nanjing Jiancheng Bioengineering Institute, Nanjing, China) and an ELISA kit (Shanghai Yuanju Biotechnology Center, Shanghai, China) according to the manufacturer’s instructions.

### 2.4 Detection of antioxidant indicators

Commercial kits (Nanjing Jiancheng Bioengineering Institute, Nanjing, China) were used to quantify superoxide dismutase (SOD), glutathione peroxidase (GSH-Px), catalase (CAT) activity, total antioxidant capacity (T-AOC) vitality, and malondialdehyde (MDA) content. All procedures were performed strictly according to the manufacturer’s instructions.

### 2.5 Detection of inflammatory indicators

Serum levels of interleukin-1β (IL-1β), interleukin-2 (IL-2), interleukin-6 (IL-6), and interleukin-10 (IL-10) were quantified with ELISA kits (Shanghai Yuanju Biotechnology Center, Shanghai, China) according to the manufacturer’s instructions.

### 2.6 GCLC, Keap1, and Nrf2 protein expression

The supernatants of the kidney homogenates were collected, and protein concentrations were determined with a BCA Protein Assay Kit (Beyotime Bio, Beijing, China). Protein expression of glutamate-cysteine ligase catalytic subunit (GCLC) and Kelch-like ECH-associated protein 1 (Keap1) was determined using the Western blot. A total of 40 μg of protein was separated on 10% sodium dodecyl sulfate–polyacrylamide gels and was subjected to electrophoresis and transferred to polyvinylidene difluoride (PVDF) membranes. The PVDF membranes were incubated overnight with primary antibodies to 1:5,000 GCLC (12601-1-AP, Proteintech Group), 1:2,000 Keap1 (80744-1-RR, Proteintech Group), and 1:5,000 Nrf2 (A69158, Hangzhou Huidan Biotechnology Co, Ltd., Hangzhou, China) on a shaker at 4 °C. The membranes were then incubated with anti-rabbit horseradish peroxidase-conjugated 1:5,000 IgG (SA00001-2, Proteintech Group, Chicago, USA) for 2 h at room temperature (14). Then, immunoreactivity was detected with an enhanced chemiluminescence reaction. The density of the bands was quantified by Image J version 2.0 (USA).

### 2.7 Kidney histopathological observation

After fixation in 10% neutral-buffered formalin for 24 h, kidney specimens were dehydrated through a graded ethanol series, cleared in xylene, and embedded in paraffin. Serial 4 μm sections were cut on a rotary microtome, mounted on glass slides, and stained with the hematoxylin and eosin method. Slides were covered with mounting medium and examined under a light microscope. To assess pathological alterations, a trained and blinded pathologist inspected the sections. A scoring system (ranging from 0 to 4 points), based on the extent of injury (< 25% injury, 25–50% injury, 50–75% injury, and > 75% injury), was used to gauge the severity of glomerular sclerosis through PAS staining.

### 2.8 The ultrastructural changes of kidney tissue

Kidney cortex samples (1 mm^3^) were excised from each group and immediately immersed in ice-cold 2.5% glutaraldehyde for 2 h. After two rinses with 0.2 M phosphate buffer (pH 7.4), the tissue was post-fixed with 1% osmium tetroxide for 2 h, washed twice more with the same buffer. Specimens were dehydrated through graded ethanol solutions, infiltrated with epoxy resin (Epon 812), and polymerized at 60 °C for 48 h. Ultrathin sections (70 nm) were cut on an ultramicrotome, mounted on copper grids, and contrast-stained with uranyl acetate followed by lead citrate. Ultrastructural changes in kidney tubular epithelial cells were examined using a transmission electron microscope.

### 2.9 Data statistical analysis

All data are expressed as the mean ± standard deviation from at least three independent experiments. The results were analyzed using one-way analysis of variance followed by the LSD test (SPSS 20.0 software; SPSS Inc., Chicago, IL, USA). The histograms were drawn by GraphPad Prism (version 8.0, GraphPad Software Inc., San Diego, CA, USA). Statistical significance was indicated as follows: ***p < 0.001, highly significant; **p < 0.01, markedly significant; *p < 0.05, significant; and ns *p* > 0.05, not significant. SD values are shown above each column in the graphs.

## 3 Results

### 3.1 Analysis of renal biomarkers: Cr, BUN, and Kim‑1

**Figure 1:**
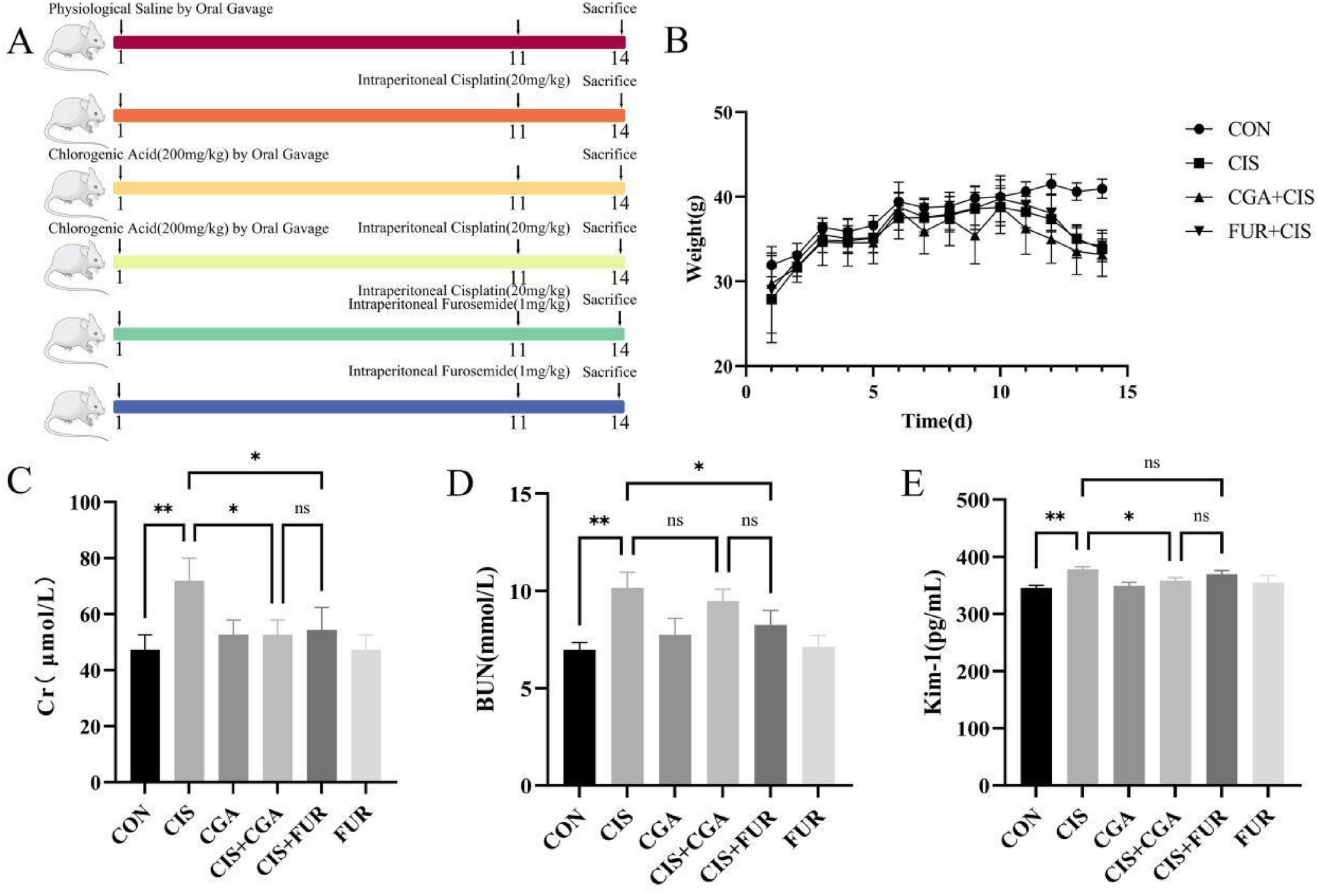
Kidney function indicators of mice (*n* = 10 per group). **(A)** represents the experimental plan, **(B)** represents the 14-day longitudinal trajectory of mouse body weight, **(C)** represents the Cr level, **(D)** represents the BUN level, and **(E)** represents the KIM-1 level. All data are expressed as the mean ± standard deviation. The results were analyzed using one-way analysis of variance followed by the LSD test (SPSS 20.0 software; SPSS Inc., Chicago, IL, USA). The histograms were drawn by GraphPad Prism (version 8.0, GraphPad Software Inc., San Diego, CA, USA). **p < 0.01, *p < 0.05, and ns *p* > 0.05. SD values were shown above each column in the graphs.

The animal protocol is shown in Figure 1A. As shown in Figure 1, the Cr level in the CIS + CGA group showed a significant decrease (*p* < 0.05, Figure 1C). Although the BUN level also decreased, the change was not statistically significant (*p* > 0.05) (Figure 1D). In contrast, the KIM-1 level declined significantly (*p* < 0.05) (Figure 1E). The levels of Cr, BUN, and KIM-1 showed no significant difference between the CIS + CGA group and the CIS + FUR group (*p* > 0.05). It suggests that preventive CGA supplementation has a protective effect on AKI. Concomitantly, serum Cr, BUN, and KIM-1 levels increased significantly. The progressive decline in body weight confirmed successful AKI induction (Figure 1B).

### 3.2 Antioxidant indicators in mice

**Figure 2:**
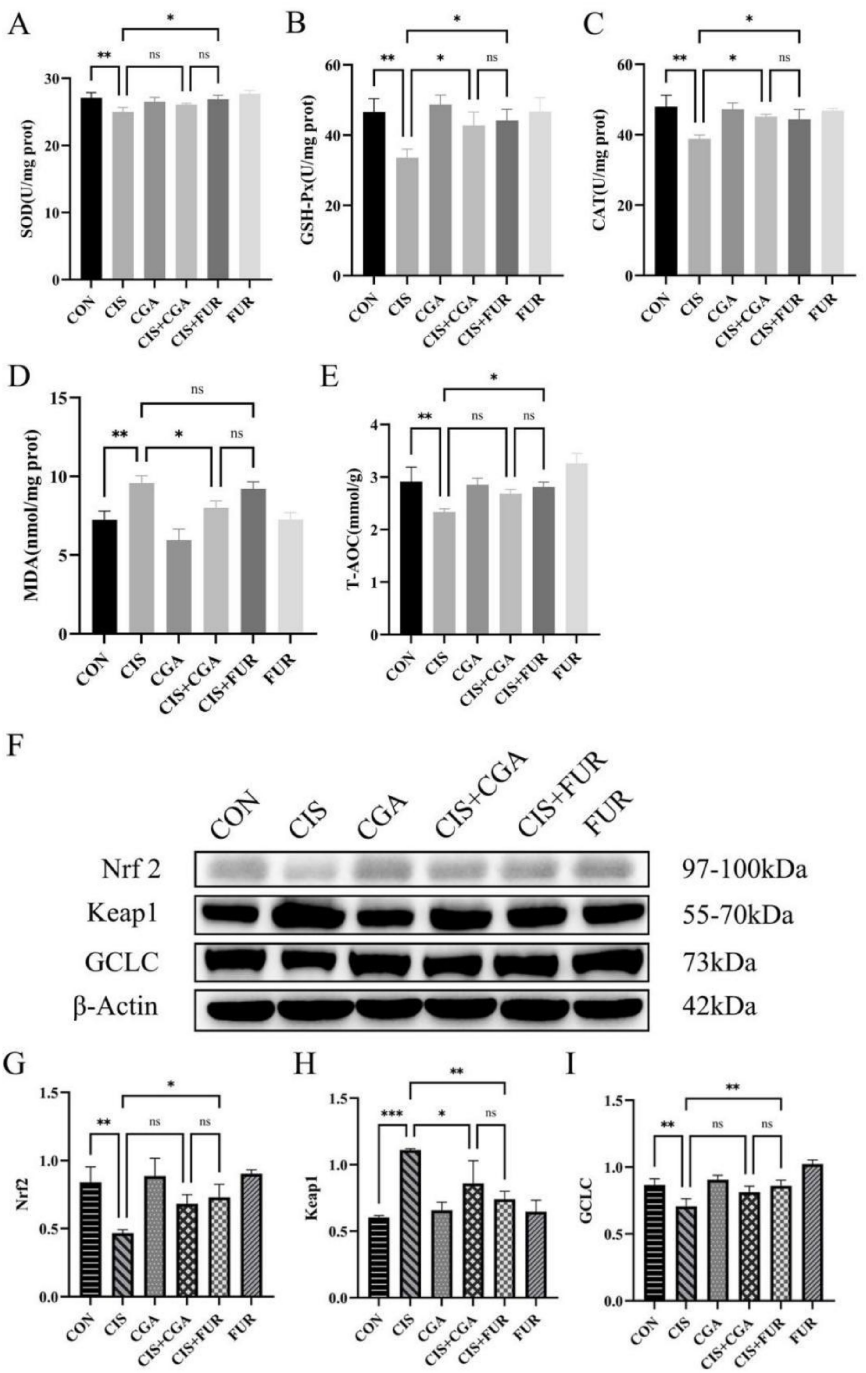
Antioxidant indicators of mice (*n* = 10 per group). **(A)** SOD activity, **(B)** GSH-Px activity, **(C)** CAT activity, **(D)** MDA level, **(E)** T-AOC vitality, **(F)** Western blot results of Keap1, Nrf2, and GCLC. **(G)** the expression of Nrf2 protein, **(H)** the expression of Keap1 protein **(I)**, and the expression of GCLC protein. All data are expressed as the mean ± standard deviation. The results were analyzed using one-way analysis of variance followed by the LSD test (SPSS 20.0 software; SPSS Inc., Chicago, IL, USA). The histograms were drawn by GraphPad Prism (version 8.0, GraphPad Software Inc., San Diego, CA, USA). *p < 0.001, **p < 0.01, *p < 0.05, and ns *p* > 0.05. SD values were shown above each column in the graphs.

In the CGA + CIS group, SOD activity did not significantly increase (*p* > 0.05) (Figure 2A). GSH-Px and CAT activities were significantly elevated (*p* < 0.05) (Figures 2B,C). MDA levels were markedly reduced (*p* < 0.05) (Figure 2D), and total antioxidant capacity (T-AOC) vitality displayed a slight, non-significant increase (*p* > 0.05) (Figure 2E). The protein expression of Nrf2 and GCLC was increased (*p* > 0.05), whereas Keap1 expression was significantly decreased (*p* < 0.05) (Figures 2F–I). No significant differences in antioxidant indices or protein levels were observed between the CIS + CGA and CIS + FUR groups (*p* > 0.05). It indicated that preventative CGA supplementation enhanced the antioxidant capacity of the mouse kidneys.

### 3.3 Analysis of inflammatory cytokines (IL-1β, IL-2, IL-6, IL-10)

**Figure 3:**
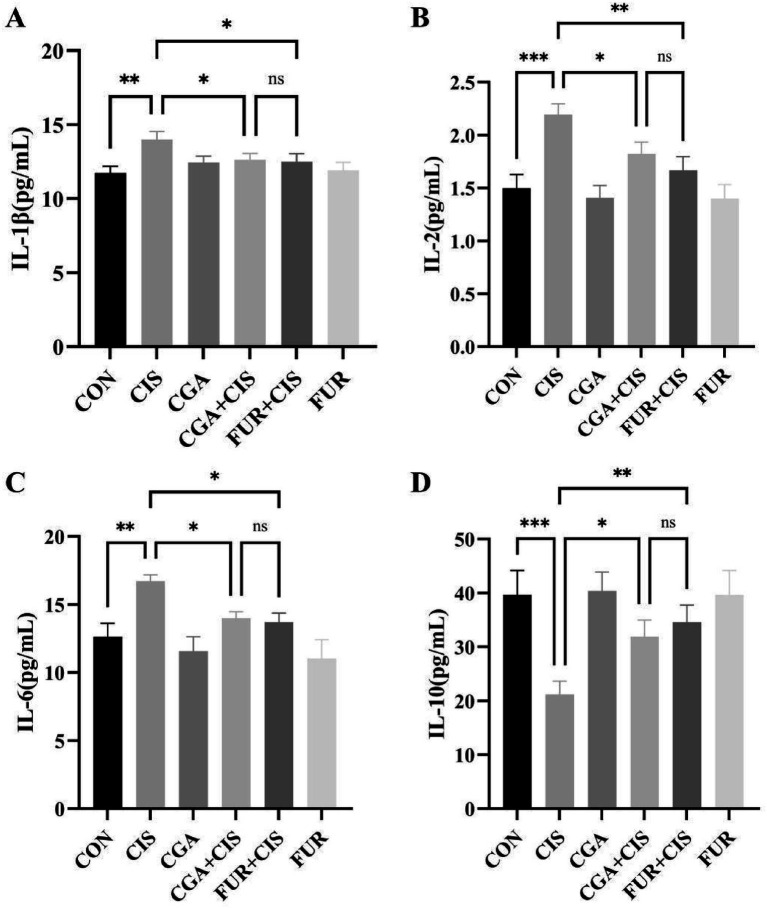
Inflammatory indicators in mice (*n* = 10 per group). **(A)** The level of IL-1β, **(B)** the level of IL-2, **(C)** the level of IL-6, **(D)** the level of IL-10. All data are expressed as the mean ± standard deviation. The results were analyzed using one-way analysis of variance followed by the LSD test (SPSS 20.0 software; SPSS Inc., Chicago, IL, USA). The histograms were drawn by GraphPad Prism (version 8.0, GraphPad Software Inc., San Diego, CA, USA). ***p < 0.001, **p < 0.01, *p < 0.05, and ns *p* > 0.05. SD values were shown above each column in the graphs.

As shown in Figure 3, the levels of IL-1β, IL-2, and IL-6 in the CIS + CGA group were significantly reduced (*p* < 0.05, Figures 3A–C), while the level of IL-10 was significantly increased (*p* < 0.05) (Figure 3D). No significant differences (*p* > 0.05) in the levels of IL-1β, IL-2, IL-6, and IL-10 were found between the CIS + CGA group and the CIS + FUR group. These findings suggest that preventive CGA supplementation effectively mitigates renal inflammatory responses.

### 3.4 Histopathological observation of the kidney

**Figure 4:**
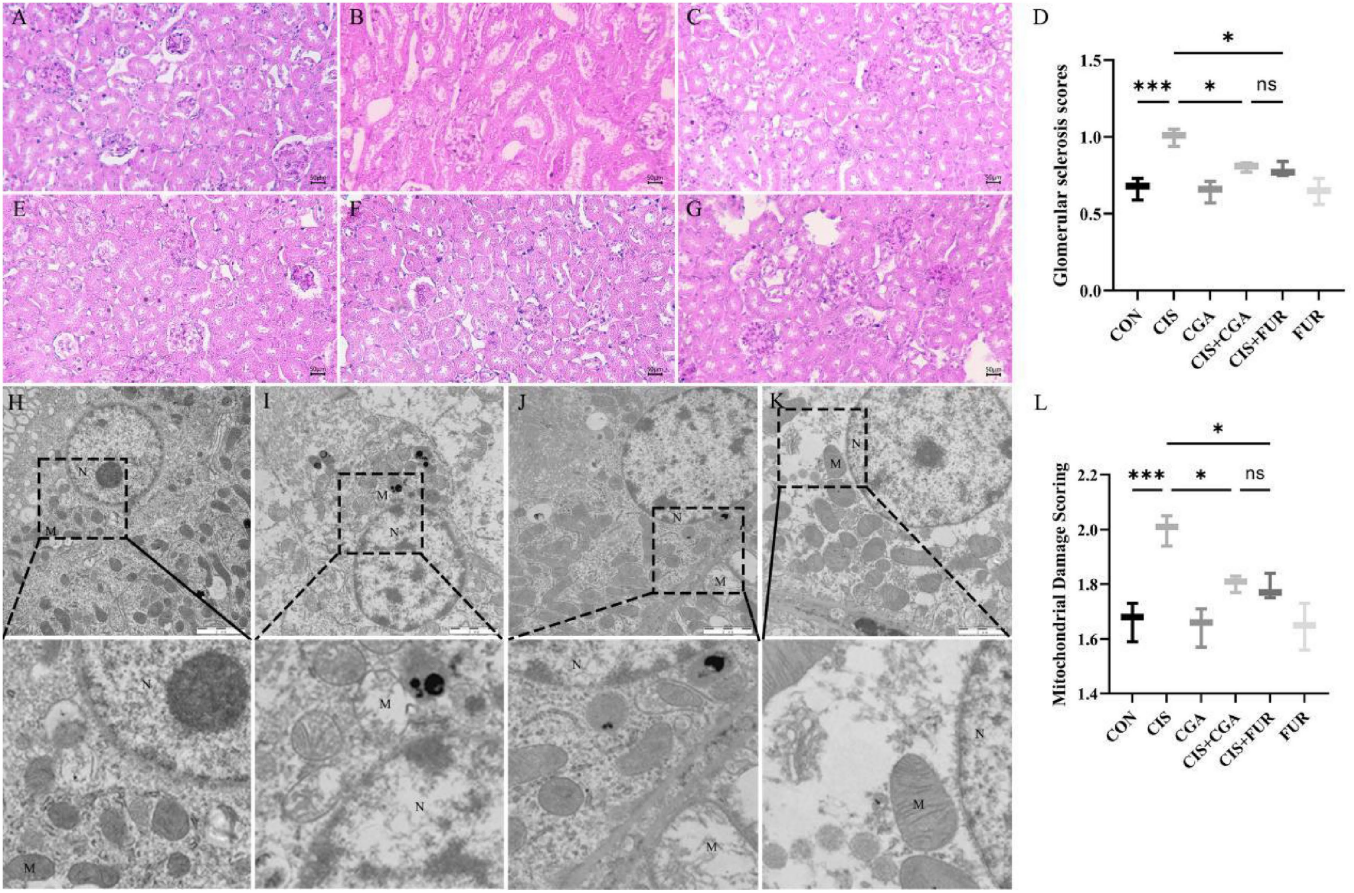
Histopathological observation (50 μm) and ultrastructural changes (*n* = 10 per group, 2 μm) of kidney tissues in mice. **(A,H)** CON group, **(B,I)** CIS group, **(C)** CGA group, **(D)** Glomerular sclerosis scores, **(E,J)** the CIS + CGA group, **(F,K)** the CIS + FUR group, **(G)** the FUR group, and **(L)** mitochondrial damage scoring. The arrows highlight glomerular inflammatory infiltration and intracellular proliferation. M represents mitochondria. N represents the nucleus. ***p < 0.001, **p < 0.01, *p < 0.05, and ns *p* > 0.05. SD values were shown above each column in the graphs.

As shown in Figure 4B, histological analysis of glomeruli indicated that the CIS group exhibited a higher number of inflammatory cells and increased glomerular sclerosis scores compared to the control group, partially restored by CGA. Compared with the CIS group, the CGA + CIS and FUR + CIS groups had lower inflammatory cells and glomerular sclerosis scores (Figures 4D–F). These findings suggested that CGA could attenuate CIS-induced kidney damage in mice. As shown in Figure 4I, in the CIS group, severe mitochondrial swelling and extensive disruption of cristae were present. The CIS + CGA and CIS + FUR groups exhibited largely intact mitochondrial structures, although minor cytoplasmic loss was noted in the CIS + FUR group (Figures 4J,K). Quantitative analysis of mitochondrial damage indicated that the CIS group exhibited increased mitochondrial damage scores compared to the control group, partially restored by CGA (Figure 4L). These findings indicated that preventive CGA supplementation attenuated CIS-induced mitochondrial damage.

## 4 Discussion

CGA exerts anti-inflammatory, antioxidant, and hypoglycemic effects (15). In this study, preventive CGA supplementation reduced glomerular and mitochondrial damage, augmented intrinsic antioxidant defenses, and attenuated inflammatory mediator levels in CIS-induced nephrotoxicity. D-galactose-induced renal injury primarily represents chronic renal injury, which is induced by long-term oxidative stress or metabolic disorders and leads to cellular senescence (16). Despite the differing mechanisms of injury in these two models, CGA has elevated antioxidant enzyme activities and reduced lipid peroxidation products in the D-galactose-induced kidney injury model. The D-galactose-induced kidney injury model is characterized as a senescent-type kidney injury (17), whereas the CIS-induced model serves as an AKI model (18). CIS-induced renal injury is acute, directly causing DNA damage and mitochondrial dysfunction (19). CIS and D-galactose induce renal injury via distinct mechanisms. The modeling period of D-galactose is relatively long, whereas CIS only requires a single intraperitoneal injection. Thus, CIS was chosen to build the AKI mice model. In this experiment, the AKI model was evaluated for BUN, CR, KIM-1, renal tissue damage, and ultrastructural damage. A progressive decrease in body weight, T-AOC vitality, and antioxidative enzymes (SOD, GSH-Px, and CAT), along with increased glomerular sclerosis and mitochondrial damage scores, a significant increase in kidney function biomarkers (Cr, BUN, KIM-1), and higher levels of the oxidative stress marker (MDA), were observed. It shows that the AKI mice model was successfully built and provides the basis for research on preventive CGA supplementation. Feng et al. indicate that a dose of 200 mg/kg of CGA has strong antioxidant and anti-inflammatory capabilities (12). The effect of CGA (200 mg/kg) on the liver in improving lipid metabolism in mice is also very beneficial (20). Thus, in this experiment, a dose of 200 mg/kg CGA was selected. In pharmacological research, 14 days is often regarded as the golden time point for short-term experiments. In ischemia–reperfusion injury models, the administration period of CGA is usually set at 7–14 days (21). A 14-day administration period in this experiment is sufficient to observe the preventive effect of CGA on AKI.

In this study, Cr, BUN, and KIM-1 served as key biomarkers to assess the nephroprotective efficacy of CGA. Circulating Cr levels provided a reliable index of glomerular filtration rate and overall kidney excretory function (22). BUN is the most widely used biomarker for kidney function assessment (23). KIM-1 is often used as an early biomarker for the prompt detection of kidney injury (24). In this experiment, preventive CGA and FUR supplementation reduced serum Cr, BUN, and KIM-1 in the CGA + CIS group. It indicated that glomerular filtration function was restored by preventive CGA and FUR supplementation. It was further supported by the reduction in scores of glomerular sclerosis (HE) and mitochondrial damage (TEM). When glomerular filtration was restored, Cr, BUN, and KIM-1 excretion increased, and kidney clearance of BUN was alleviated, causing the reduction of their serum concentration. It indicates that preventive CGA supplementation mitigates CIS-induced AKI. In a sodium arsenite (NaAsO_2_)-induced murine model of nephrotoxicity, Al-Megrin WA (25) found that CGA markedly lowered serum Cr and BUN, affirming its reno-protective capacity. It demonstrates the early kidney-protective efficacy of CGA. However, in this study, there was no difference between CGA and FUR in the key biomarkers of kidney function (Cr, BUN, and KIM-1). It is necessary to enlarge the experiment period to determine the difference between CGA and FUR.

Oxidative stress is a common pathological mechanism underlying various kidney diseases. The mechanisms of CGA involve the modulation of oxidative stress. GSH-Px, SOD, and CAT were key enzymes in the metabolism of H_2_O_2_ and reactive nitrogen species (26). Total antioxidant capacity (T-AOC) is a biomarker often used to investigate oxidative stress under many pathological conditions (27). MDA content served as a key biomarker reflecting systemic antioxidant status. In this experiment, preventive CGA and FUR supplementation increased the enzymatic activities of SOD, GSH-Px, and CAT, while concurrently decreasing the MDA content. In a D-galactose-induced murine model of kidney injury, CGA restored kidney levels of SOD, CAT, and GSH activity, while lowering the MDA content (12). CGA could directly scavenge free radicals (15). This suggests that CGA can mitigate renal damage by modulating oxidative stress levels, thereby providing valuable data to support further investigations into CGA in the context of AKI. It indicates that CGA fortifies kidney tissue against oxidative injury. In this study, there was no difference between CGA and FUR on the key biomarkers of oxidative stress (SOD, GSH-PX, CAT, T-AOC). It may be the short period of preventive CGA supplementation. CGA upregulated the SOD, CAT, and GSH-Px activities in LPS-treated cells (28). This finding contrasts with our present results.

In this experiment, preventive CGA and FUR supplementation increased the protein expression of Nrf2 and GCLC, and reduced the protein expression of Keap1. Nrf2, a major transcription factor, regulates cellular antioxidant defense pathways. During AKI, Nrf2 maintains intracellular redox homeostasis, effectively attenuating tubular injury and interstitial fibrosis. The increase in Nrf2 may promote the expression of antioxidant enzymes, such as CAT, SOD, and GSH (29). KEAP1 is an adaptor subunit of CULLIN 3 (CUL3)-based E3 ubiquitin ligase. Keap1 regulates the activity of Nrf2 and acts as a sensor of oxidative and electrophilic stresses (30). Keap1 acted as a negative regulator of Nrf2, suppressing Nrf2 protein expression under physiological conditions. The concurrent increase in Nrf2 and decrease in Keap1 support the hypothesis, although their interaction was not examined. The Keap1-Nrf2 signaling pathway is essential for controlling cellular defense mechanisms against oxidative stress (31). Further experiments will be conducted to explore the effects of preventive CGA and FUR supplementation on the Keap1-Nrf2 signaling pathway. Previous studies have shown that reduced Keap1 levels are associated with decreases in Cr, BUN, and KIM-1 levels in mice, further supporting its protective role in the kidney (32). Our results also found a reduction in serum Cr, BUN, and KIM-1 in the CGA + CIS group. It is in line with the evidence. Additionally, evidence indicates that Nrf2 directly regulates GCLC, the rate-limiting enzyme in glutathione synthesis. GCLC enhanced intracellular non-enzymatic antioxidant capacity and alleviated oxidative stress-induced damage (33). The results of this experiment demonstrate that preventive CGA and FUR supplementation increase the protein expression of GCLC, suggesting that preventive CGA and FUR supplementation exerts kidney protection through the modulation of the oxidative stress pathway.

IL-1, IL-2, and IL-6 are pro-inflammatory cytokines, while IL-10 exerts anti-inflammatory effects and prevents damage caused by excessive inflammation (34). This study demonstrated that preventive CGA and FUR supplementation decreases the IL-1β, IL-2, and IL-6 levels in mice, while increasing the level of IL-10. It demonstrates its ability to suppress kidney inflammation. IL-1β and IL-6 are classic pro-inflammatory cytokines. IL-1β is the initiating factor of inflammatory responses, while IL-6 is a marker of acute reactions (35). Decreased IL-6 is often associated with recovery of tissue damage and represents an acute response of the body to kidney function. IL-2 is a key factor in T-cell proliferation and NK-cell activation (36). The significant decrease in IL-2 levels indicates the effect of preventive CGA and FUR supplementation on the immune activation state of the mouse body. IL-10 is a typical anti-inflammatory cytokine, and an elevated level of it reflects the body’s regulatory capacity for inflammatory responses. Reduced IL-10 reflects negative feedback on excessive inflammation (37). Our results indicate that CGA can significantly reduce the levels of IL-1β and IL-6 in serum, while restoring the levels of IL-10 and IL-2, suggesting that CGA has multi-faceted immunomodulatory effects. This anti-inflammatory effect is consistent with previous research results, that is, chlorogenic acid alleviates LPS-induced acute kidney injury by inhibiting the TLR4/NF-κB signaling pathway, thereby reducing the production of IL-1β and IL-6 (38). In addition, chlorogenic acid can increase the level of IL-10, which is consistent with its role in promoting anti-inflammatory responses and renal protection, as IL-10 has been shown to inhibit the expression of pro-inflammatory cytokines in CIS-induced nephrotoxicity (39). The recovery of IL-2 levels further indicates that chlorogenic acid may help to restore immune homeostasis, possibly by regulating T-cell activity. Based on histopathological examination of kidney tissue, kidneys from the CGA + CIS group, which received preventive CGA supplementation, showed attenuated CIS kidney damage, supporting the conclusion that CGA mitigated kidney injury by anti-inflammation activity.

## 5 Conclusion

These results indicate that preventive CGA supplementation effectively mitigates CIS-induced AKI by enhancing antioxidant capacity, attenuating inflammatory responses, and alleviating kidney structural damage. This study provides pivotal evidence supporting the potential use of CGA in AKI prevention.

## Data Availability

The original contributions presented in the study are included in the article/Supplementary material, further inquiries can be directed to the corresponding author/s.

## References

[1] Li W Xiang Z Xing Y Li S Shi S. Mitochondria bridge HIF signaling and ferroptosis blockage in acute kidney injury. Cell Death Dis. (2022) 13:308. doi: 10.1038/s41419-022-04770-4, 35387983 PMC8986825

[2] Liu J Li Q Lai D Chen G Wang B Liu L . Trends in incidence and long-term prognosis of acute kidney injury following coronary angiography in Chinese cohort with 11,943 patients from 2013 to 2017: an observational study. BMC Nephrol. (2021) 22:235. doi: 10.1186/s12882-021-02427-6, 34172005 PMC8235610

[3] Zhao GJ Xu C Ying JC Lü WB Hong GL Li MF . Association between furosemide administration and outcomes in critically ill patients with acute kidney injury. Crit Care. (2020) 24:75. doi: 10.1186/s13054-020-2798-6, 32131879 PMC7057586

[4] Xu FB Cheng H Yue T Ye N Zhang HJ Chen YP. Derivation and validation of a prediction score for acute kidney injury secondary to acute myocardial infarction in Chinese patients. BMC Nephrol. (2019) 20:195. doi: 10.1186/s12882-019-1379-x, 31146701 PMC6543657

[5] Caiati C Arrigoni R Stanca A Lepera ME. Kidney toxicity of drugs for the heart: an updated perspective. Meta. (2025) 15:191. doi: 10.3390/metabo15030191, 40137155 PMC11943962

[6] Jahanban-Esfahlan A Ostadrahimi A Tabibiazar M Amarowicz R. A comprehensive review on the chemical constituents and functional uses of walnut (*Juglans* spp.) husk. Int J Mol Sci. (2019) 20:3920. doi: 10.3390/ijms20163920, 31409014 PMC6719079

[7] Naveed M Hejazi V Abbas M Kamboh AA Khan GJ Shumzaid M . Chlorogenic acid (CGA): a pharmacological review and call for further research. Biomed Pharmacother. (2018) 97:67–74. doi: 10.1016/j.biopha.2017.10.064, 29080460

[8] Xia S Yu H Qiu Y Zhao Y Li H Zhang J . A novel curdlan/methyl cellulose/walnut green husk polyphenol edible composite film for walnut packaging. Int J Biol Macromol. (2024) 261:129505. doi: 10.1016/j.ijbiomac.2024.129505, 38232883

[9] Hu H Hu J Chen Z Yang K Zhu Z Hao Y . RBBP6-mediated ERRα degradation contributes to mitochondrial injury in renal tubular cells in diabetic kidney disease. Adv Sci. (2024) 11:e2405153. doi: 10.1002/advs.202405153, 39441040 PMC11633482

[10] Jiao H Zhang M Xu W Pan T Luan J Zhao Y . Chlorogenic acid alleviate kidney fibrosis through regulating TLR4/NF-қB mediated oxidative stress and inflammation. J Ethnopharmacol. (2024) 335:118693. doi: 10.1016/j.jep.2024.118693, 39142620

[11] Ertuglu L Yildiz A Gamboa J Ikizler TA. Skeletal muscle energetics in patients with moderate to advanced kidney disease. Kidney Research Clinical Practice. (2022) 41:14–21. doi: 10.23876/j.krcp.21.175, 35108768 PMC8816417

[12] Feng Y Yu YH Wang ST Ren J Camer D Hua YZ . Chlorogenic acid protects D-galactose-induced liver and kidney injury via antioxidation and anti-inflammation effects in mice. Pharm Biol. (2016) 54:1027–34. doi: 10.3109/13880209.2015.1093510, 26810301 PMC11132915

[13] Street JM Bellomo TR Koritzinsky EH Kojima H Yuen PST Star RA. A furosemide excretion stress test predicts mortality in mice after sepsis and outperforms the furosemide stress test during vasopressin administration. Crit Care Explor. (2020) 2:e0112. doi: 10.1097/CCE.0000000000000112, 32671344 PMC7259566

[14] Zhou W He H Wei Q Che L Zhao X Liu W . Puerarin protects against acetaminophen-induced oxidative damage in liver through activation of the Keap1/Nrf2 signaling pathway. Food Sci Nutr. (2023) 11:6604–15. doi: 10.1002/fsn3.3609, 37823166 PMC10563760

[15] Nguyen V Taine EG Meng D Cui T Tan W. Chlorogenic acid: a systematic review on the biological functions, mechanistic actions, and therapeutic potentials. Nutrients. (2024) 16:924. doi: 10.3390/nu16070924 38612964 PMC11013850

[16] Shi J Xu Y Zhang K Liu Y Zhang N Zhang Y . Fucoidan oligosaccharide supplementation relieved kidney injury and modulated intestinal homeostasis in D-galactose-exposed rats. Nutrients. (2025) 17:325. doi: 10.3390/nu17020325 39861454 PMC11769225

[17] Liu B Tu Y He W Liu Y Wu W Fang Q . Hyperoside attenuates renal aging and injury induced by D-galactose via inhibiting AMPK-ULK1 signaling-mediated autophagy. Aging. (2018) 10:4197–212. doi: 10.18632/aging.101723, 30585174 PMC6326678

[18] Guo S Zhou L Liu X Gao L Li Y Wu Y. Baicalein alleviates cisplatin-induced acute kidney injury by inhibiting ALOX12-dependent ferroptosis. Phytomedicine. (2024) 130:155757. doi: 10.1016/j.phymed.2024.155757, 38805781

[19] Zhang Y Qin S Song Y Yuan J Hu S Chen M . Alginate oligosaccharide alleviated cisplatin-induced kidney oxidative stress via *lactobacillus* genus-FAHFAs-Nrf2 axis in mice. Front Immunol. (2022) 13:857242. doi: 10.3389/fimmu.2022.857242, 35432359 PMC9010505

[20] Cho AS Jeon SM Kim MJ Yeo J Seo KI Choi MS . Chlorogenic acid exhibits anti-obesity property and improves lipid metabolism in high-fat diet-induced-obese mice. Food Chemical Toxicol. (2010) 48:937–43. doi: 10.1016/j.fct.2010.01.003, 20064576

[21] Yunus J Salman M Lintin GBR Muchtar M Sari DCR Arfian N . Chlorogenic acid attenuates kidney fibrosis via antifibrotic action of BMP-7 and HGF. Med J Malaysia. (2020) 75:5–9. 32471962

[22] Ávila M Mora Sánchez MG Bernal Amador AS Paniagua R. The metabolism of creatinine and its usefulness to evaluate kidney function and body composition in clinical practice. Biomolecules. (2025) 15:41. doi: 10.3390/biom15010041 39858438 PMC11764249

[23] Gong Q Lai T Liang L Jiang Y Liu F. Targeted inhibition of CX3CL1 limits podocytes ferroptosis to ameliorate cisplatin-induced acute kidney injury. Mol Med. (2023) 29:140. doi: 10.1186/s10020-023-00733-3, 37875838 PMC10594885

[24] Yang L Brooks CR Xiao S Sabbisetti V Yeung MY Hsiao LL . KIM-1-mediated phagocytosis reduces acute injury to the kidney. J Clin Invest. (2015) 125:1620–36. doi: 10.1172/JCI75417, 25751064 PMC4396492

[25] Al-Megrin WA Metwally DM Habotta OA Amin HK Abdel Moneim AE El-Khadragy M. Nephroprotective effects of chlorogenic acid against sodium arsenite-induced oxidative stress, inflammation, and apoptosis. J Sci Food Agric. (2020) 100:5162–70. doi: 10.1002/jsfa.10565, 32519758

[26] Pongsavee M. Effects of ERCC5 rs751402 polymorphism on oxidative stress and the impact of curcumin on catalase activity in breast carcinogenesis. Asian Pac J Cancer Prev. (2022) 23:2065–70. doi: 10.31557/APJCP.2022.23.6.2065, 35763649 PMC9587821

[27] Golabi S Ghasemi S Adelipour M Bagheri R Suzuki K Wong A . Oxidative stress and inflammatory status in COVID-19 outpatients: a health center-based analytical cross-sectional study. Antioxidants. (2022) 11:606. doi: 10.3390/antiox11040606, 35453291 PMC9024445

[28] Gu T Zhang Z Liu J Chen L Tian Y Xu W . Chlorogenic acid alleviates LPS-induced inflammation and oxidative stress by modulating CD36/AMPK/PGC-1α in RAW264.7 macrophages. Int J Mol Sci. (2023) 24:13516. doi: 10.3390/ijms241713516, 37686324 PMC10487601

[29] Boas SM Joyce KL Cowell RM. The NRF2-dependent transcriptional regulation of antioxidant defense pathways: relevance for cell type-specific vulnerability to neurodegeneration and therapeutic intervention. Antioxidants. (2021) 11:8. doi: 10.3390/antiox11010008, 35052512 PMC8772787

[30] Suzuki T Takahashi J Yamamoto M. Molecular basis of the KEAP1-NRF2 Signaling pathway. Mol Cells. (2023) 46:133–41. doi: 10.14348/molcells.2023.0028, 36994473 PMC10070164

[31] Liu Z Li Y Bao J Li S Wen Y Zhang P . Astaxanthin ameliorates benzalkonium chloride-induced dry eye disease through suppressing inflammation and oxidative stress via Keap1-Nrf2/HO-1 signaling pathways. Anim Models Exp Med. (2025) 8:1056–79. doi: 10.1002/ame2.70000, 40045550 PMC12205003

[32] Tan RJ Chartoumpekis DV Rush BM Zhou D Fu H Kensler TW . Keap1 hypomorphism protects against ischemic and obstructive kidney disease. Sci Rep. (2016) 6:36185. doi: 10.1038/srep36185, 27804998 PMC5090361

[33] Yoo S Kim M Bae JY Lee SA Koh G. Bardoxolone methyl inhibits ferroptosis through the Keap1-Nrf2 pathway in renal tubular epithelial cells. Mol Med Rep. (2025) 32:267. doi: 10.3892/mmr.2025.13632, 40709383 PMC12319272

[34] Al-Qahtani AA Alhamlan FS Al-Qahtani AA. Pro-inflammatory and anti-inflammatory interleukins in infectious diseases: a comprehensive review. Trop Med Infect Dis. (2024) 9:13. doi: 10.3390/tropicalmed9010013, 38251210 PMC10818686

[35] You J Zhang Y Zhou Y. Strontium functionalized in biomaterials for bone tissue engineering: a prominent role in osteoimmunomodulation. Front Bioeng Biotechnol. (2022) 10:928799. doi: 10.3389/fbioe.2022.928799, 35875505 PMC9298737

[36] D'Souza C Prince HM Neeson PJ. Understanding the role of T-cells in the antimyeloma effect of immunomodulatory drugs. Front Immunol. (2021) 12:632399. doi: 10.3389/fimmu.2021.632399, 33746969 PMC7973099

[37] Hurst CN Alexander JD Dolan BP Jia L Bartholomew JL. Outcome of within-host competition demonstrates that parasite virulence doesn't equal success in a myxozoan model system. Int J Parasitol Parasites Wildl. (2019) 9:25–35. doi: 10.1016/j.ijppaw.2019.03.008, 30976514 PMC6441732

[38] Ye HY Jin J Jin LW Chen Y Zhou ZH Li ZY. Chlorogenic acid attenuates lipopolysaccharide-induced acute kidney injury by inhibiting TLR4/NF-κB signal pathway. Inflammation. (2017) 40:523–9. doi: 10.1007/s10753-016-0498-9, 28028753

[39] Deng J Kohda Y Chiao H Wang Y Hu X Hewitt SM . Interleukin-10 inhibits ischemic and cisplatin-induced acute renal injury. Kidney Int. (2001) 60:2118–28. doi: 10.1046/j.1523-1755.2001.00043.x 11737586

