# Correction: The preventive effect of chlorogenic acid on cisplatin-induced acute kidney injury in mice

**DOI:** 10.3389/fvets.2026.1845799

**Published:** 2026-04-29

**Authors:** Zheng Hongya, Duan Yichang, Zhong Zheng, Zhu Yanzhu, Niu Wei, Huang Caoxing, Yin Baishuang

**Affiliations:** 1 College of Animal Science and Technology, Heilongjiang Bayi Agricultural University, Daqing, China; 2 Key Lab of Preventive Veterinary Medicine in Jilin Province, College of Animal Science and Technology, Jilin Agricultural Science and Technology University, Jilin, China; 3 College of Chemical Engineering, Nanjing Forestry University, Nanjing, China

**Keywords:** acute kidney injury, antioxidant capacity, chlorogenic acid, cisplatin, mice

Affiliation “Key Lab of Preventive Veterinary Medicine in Jilin Province, College of Animal Science and Technology, Jilin Agricultural Science and Technology University, Jilin, China” was omitted for author Yin Baishuang. This affiliation has now been added for author Yin Baishuang.

The original version of this article has been updated.

