# Supplementary material for: The preventive effect of chlorogenic acid on cisplatin-induced acute kidney injury in mice
Source: Front Vet Sci. 2026 Feb 19;13:1763548. doi: 10.3389/fvets.2026.1763548 (PMC12960156; doi:10.3389/fvets.2026.1763548)
Supplement: Supplementary file 1 [file Table_1.DOCX]

**western blot**

**Nrf2**

**-(1)-for Fig 2F**

**
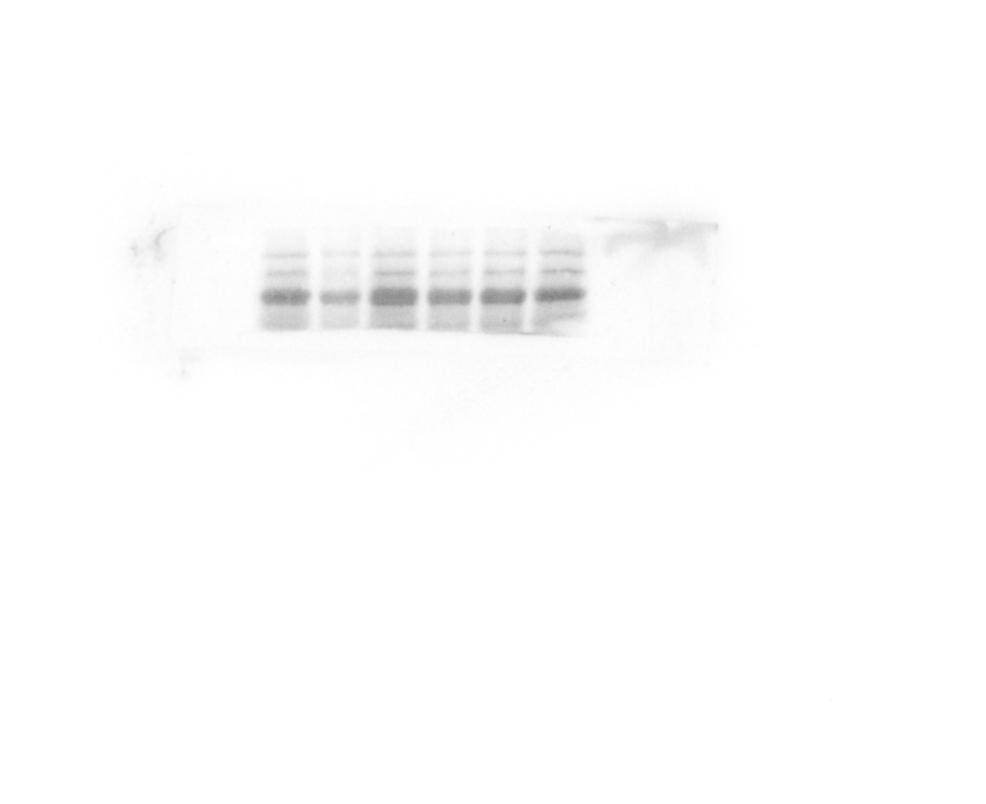

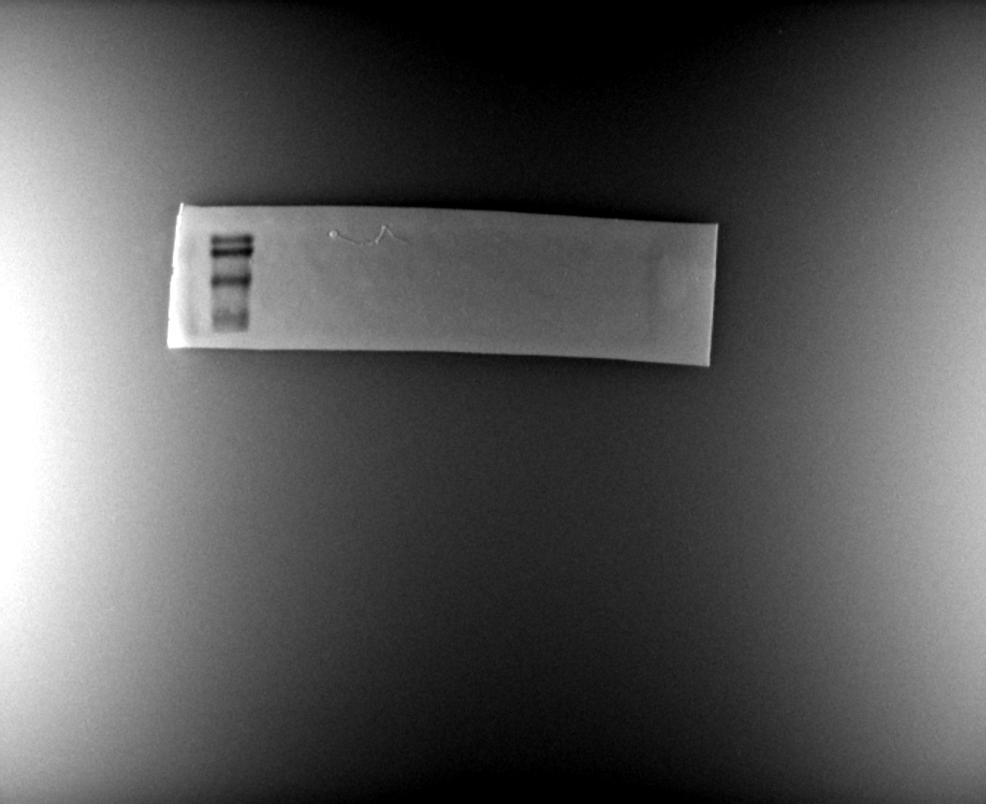
**

**97-100kDa**

**Nrf2**

**-(2)-for Fig 2F**

**
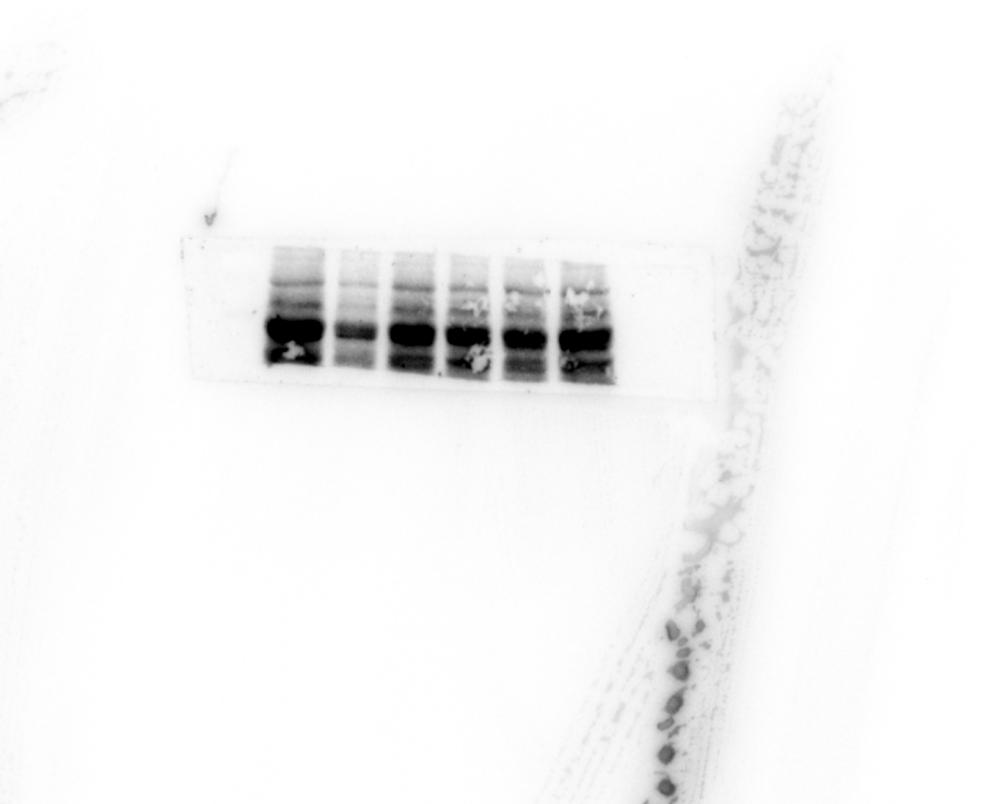

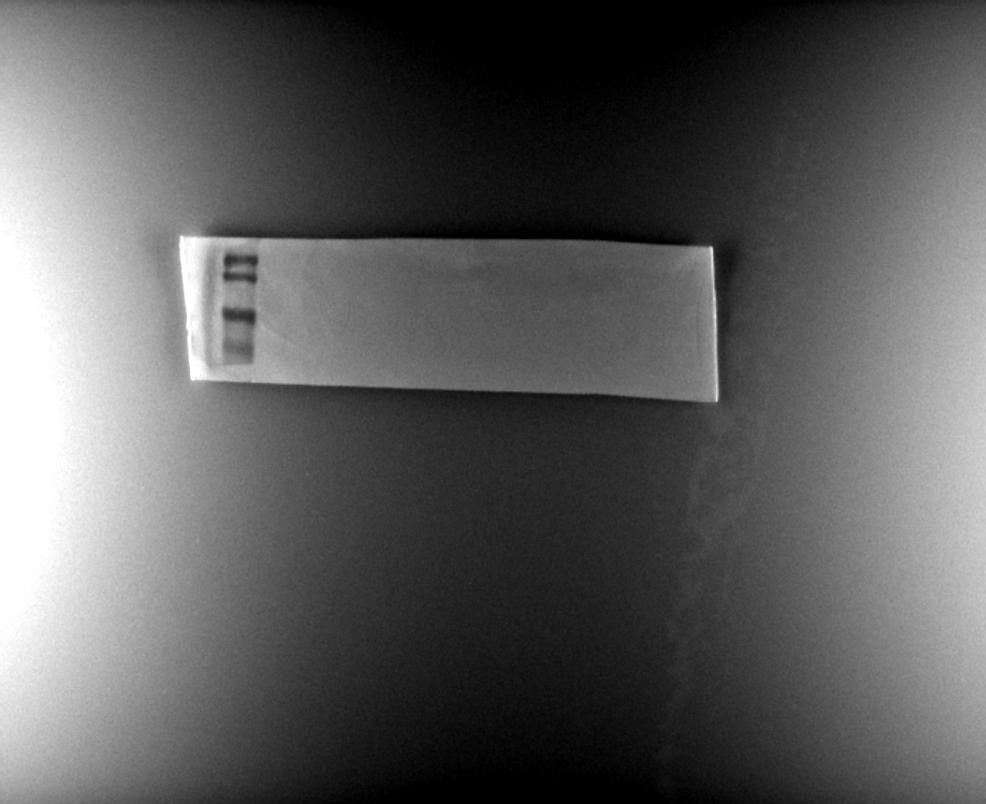
**

**97-100kDa**

**Nrf2**

**-(3)-for Fig 2F**

**
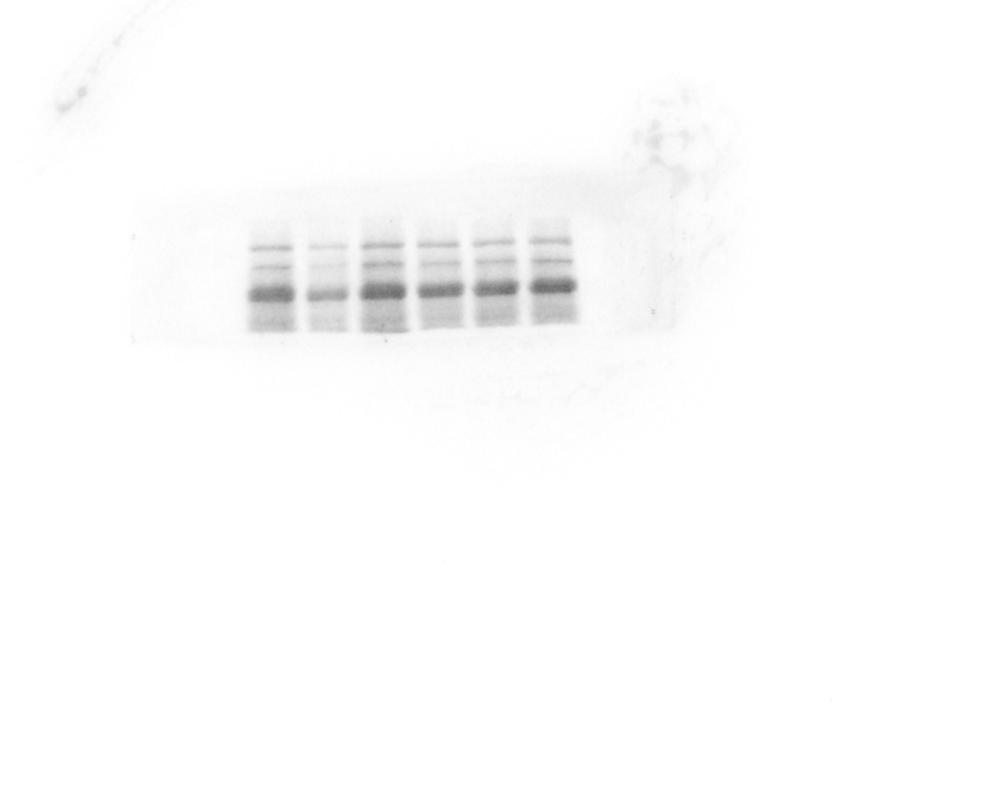

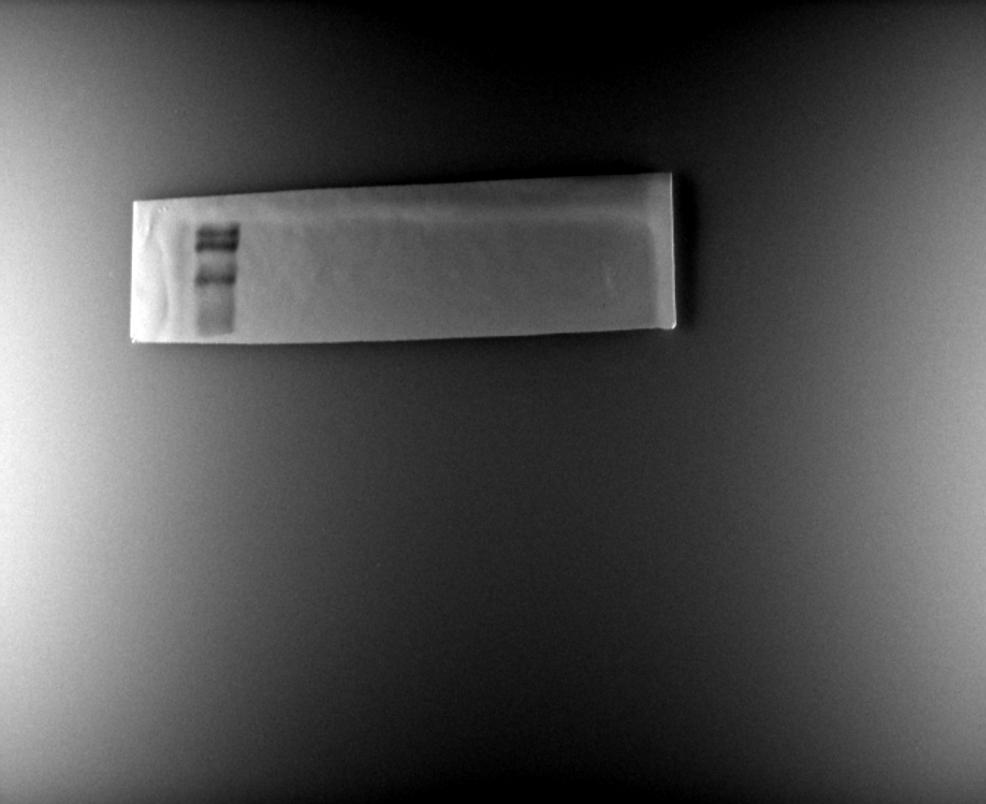
**

**97-100kDa**

**Keap1**

**-(1)-for Fig 2F**

**
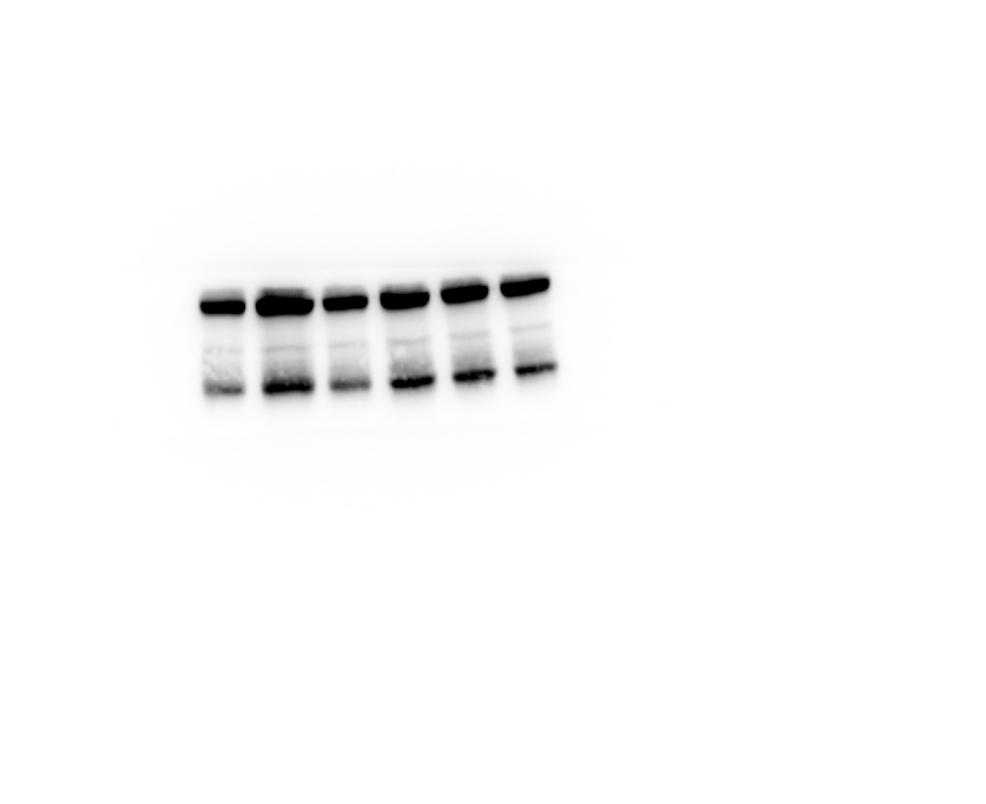

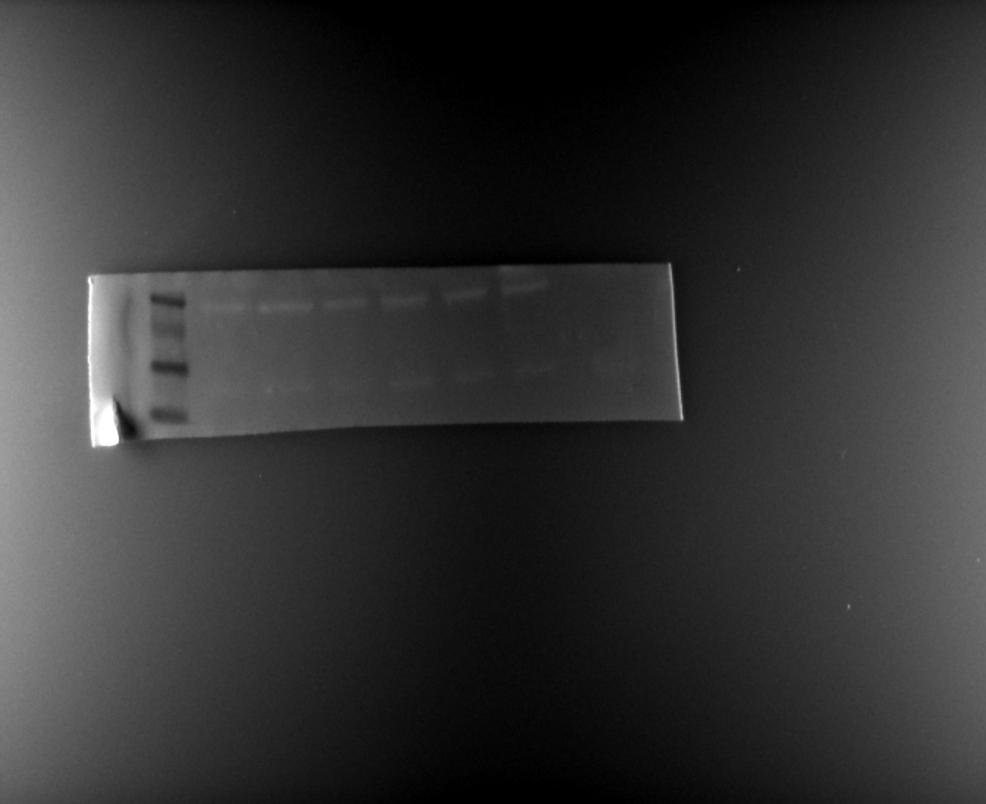
**

**55-70kDa**

**Keap1**

**-(2)-for Fig 2F**

**
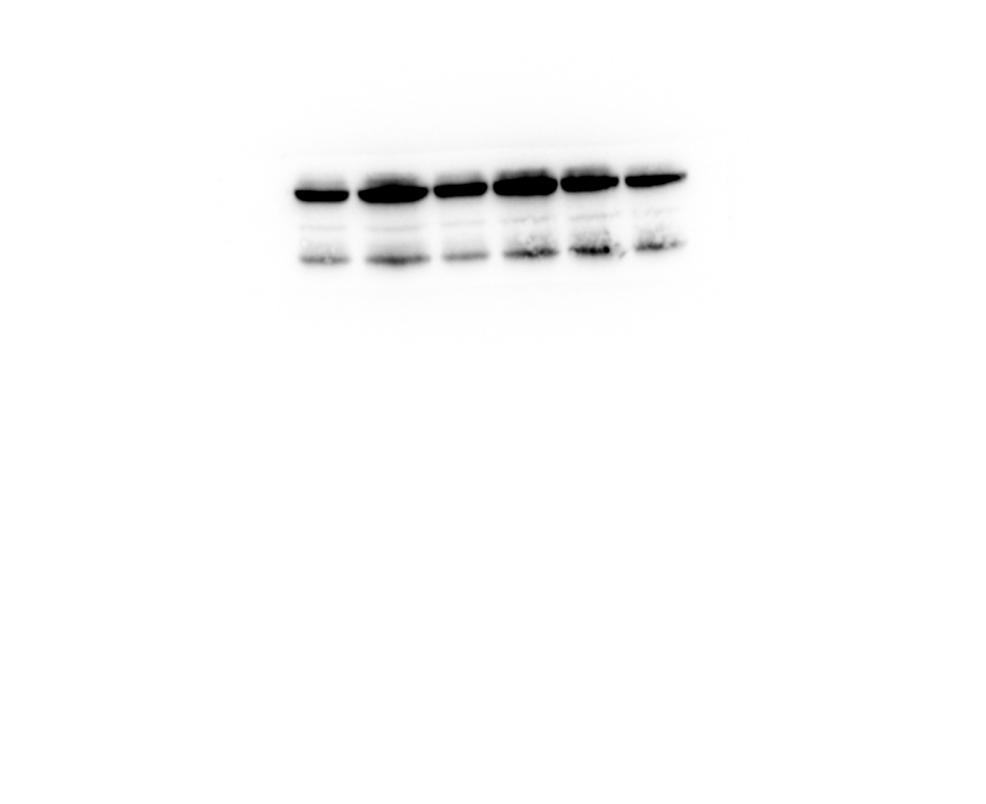

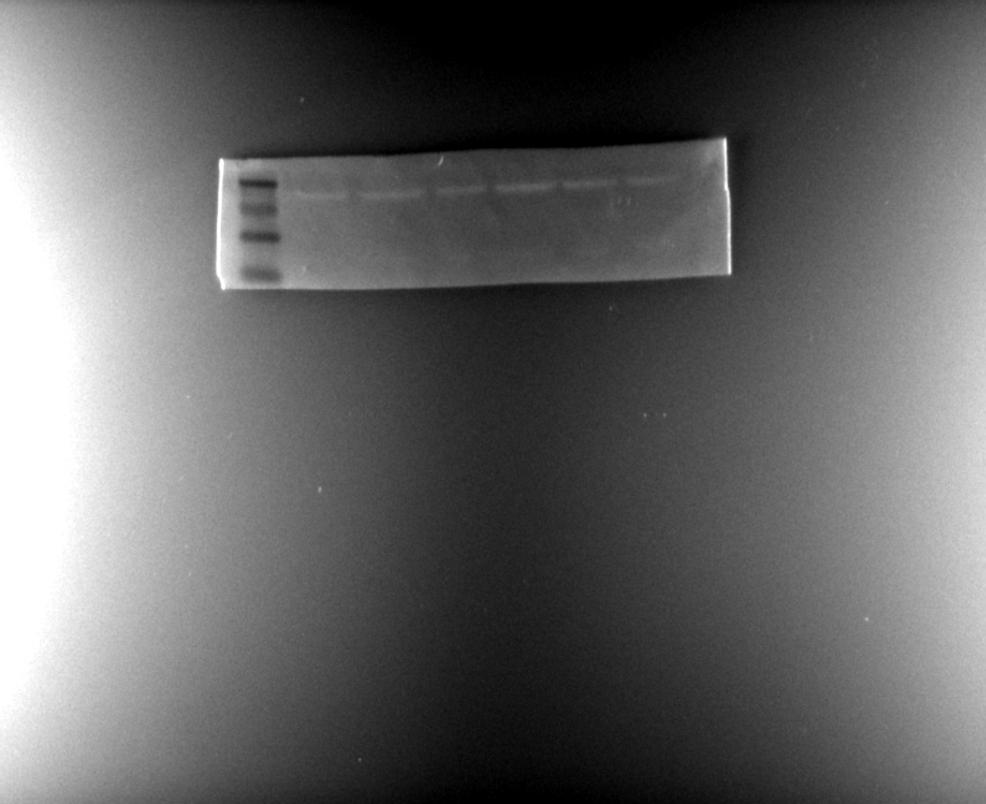
**

**55-70kDa**

**Keap1**

**-(3)-for Fig 2F**

**
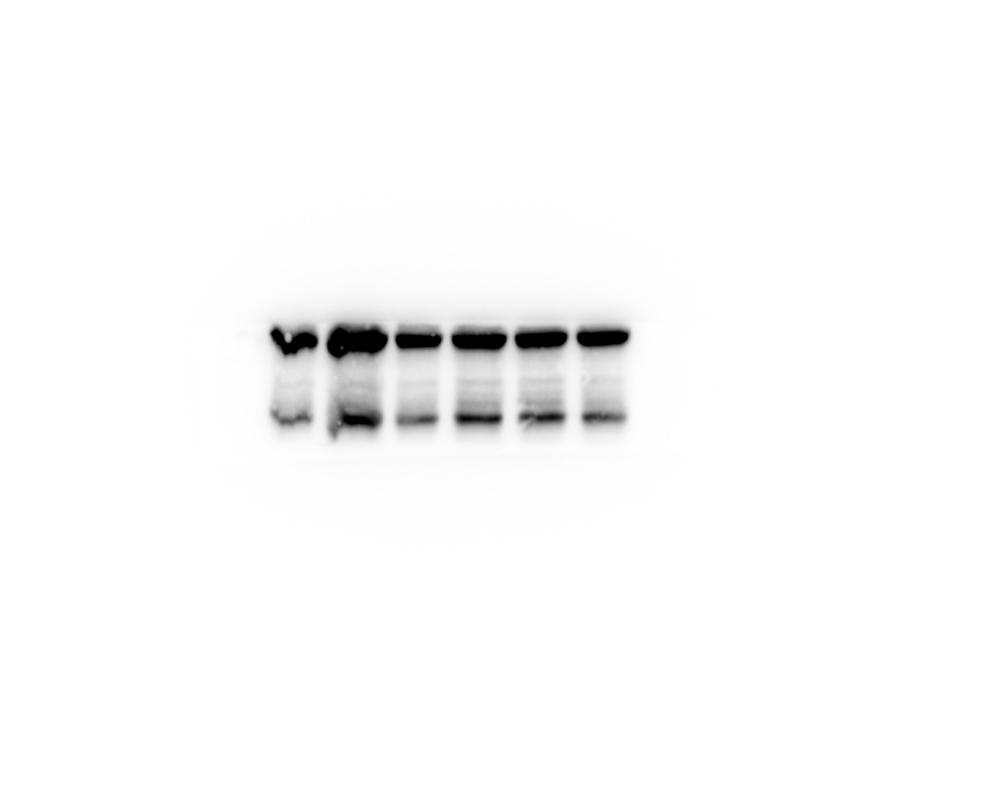

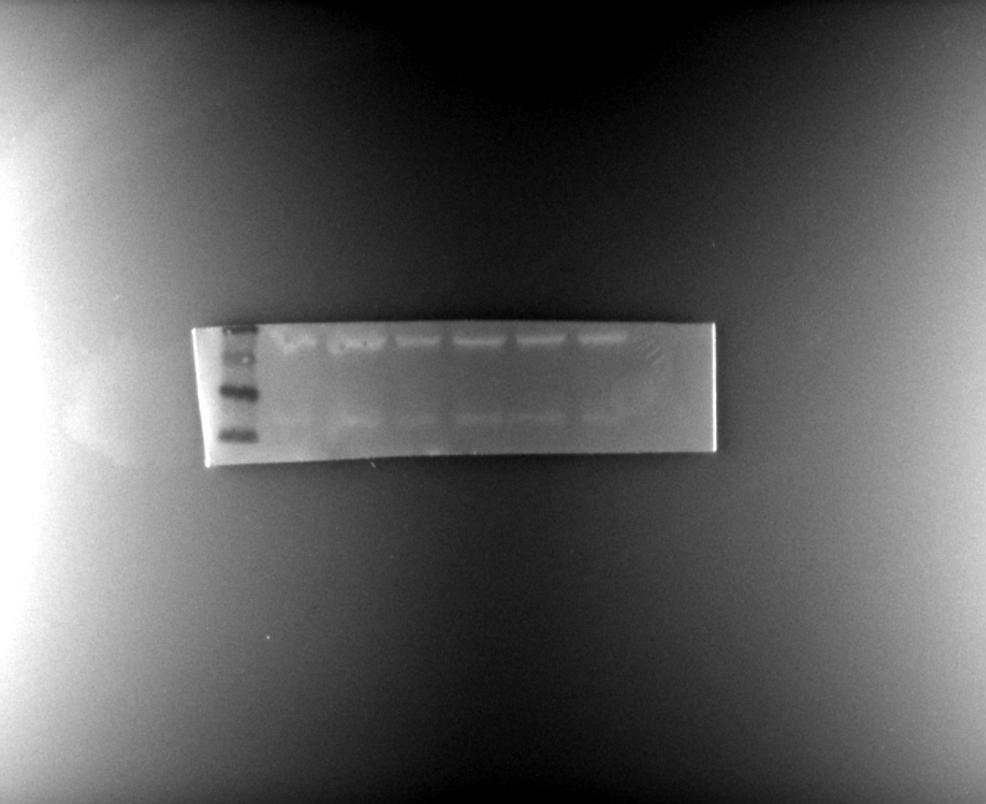
**

**55-70kDa**

**GCLC**

**-(1)-for Fig 2F**

**
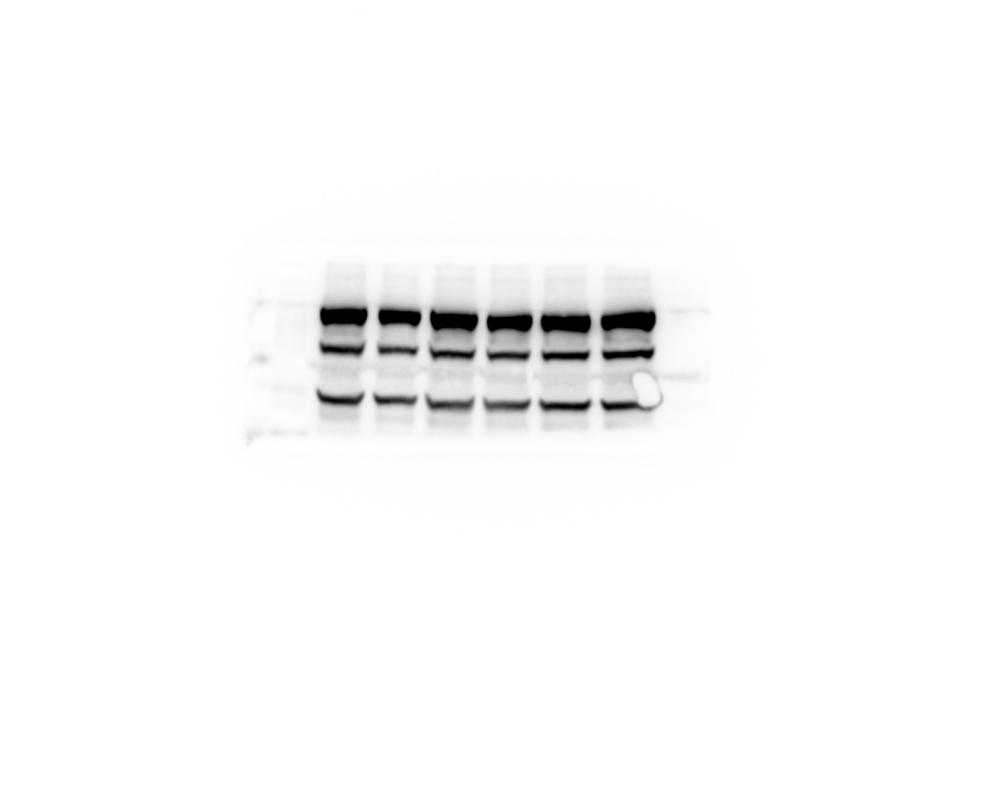

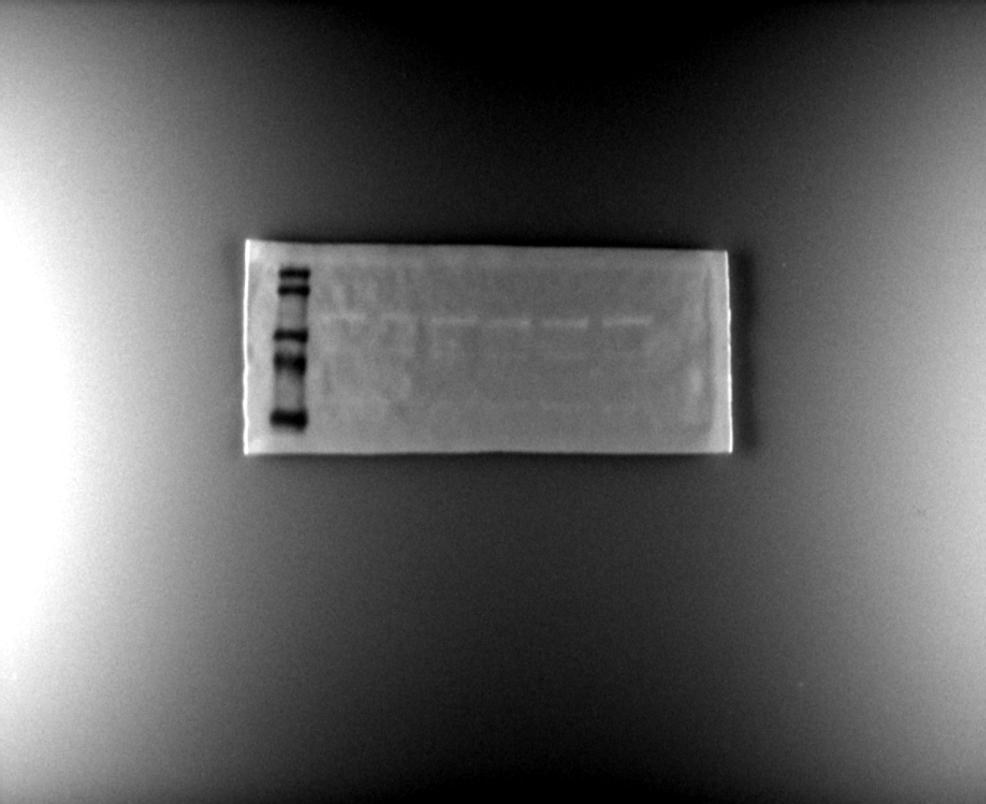
**

**73kDa**

**GCLC**

**-(2)-for Fig 2F**

**
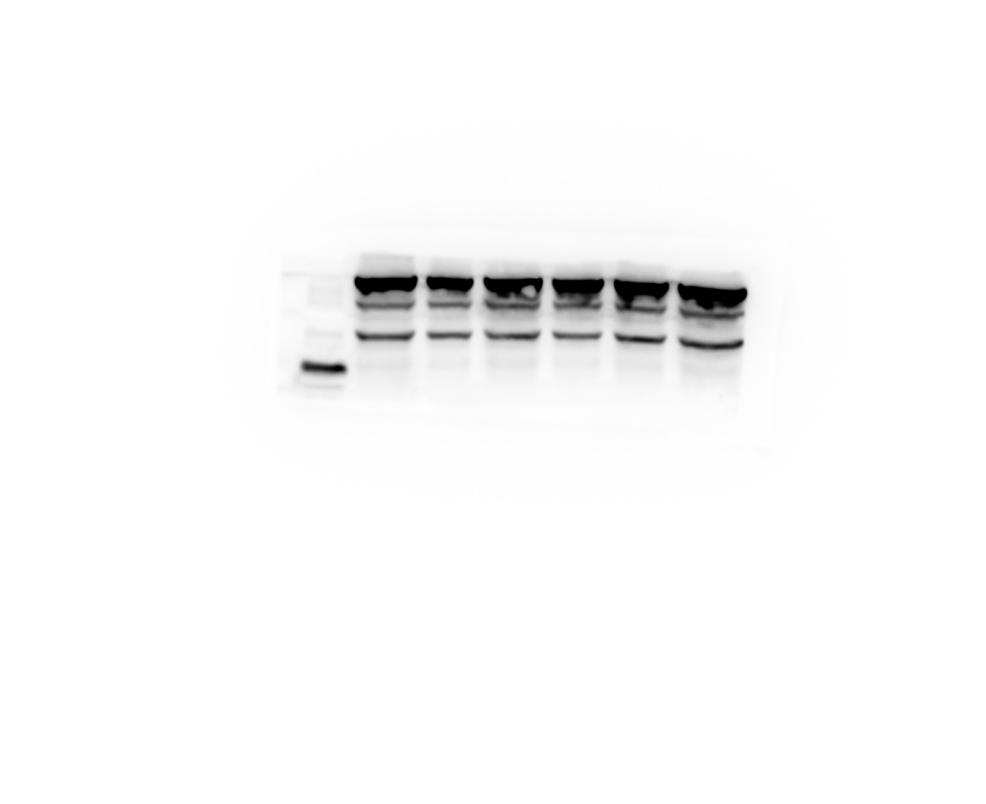

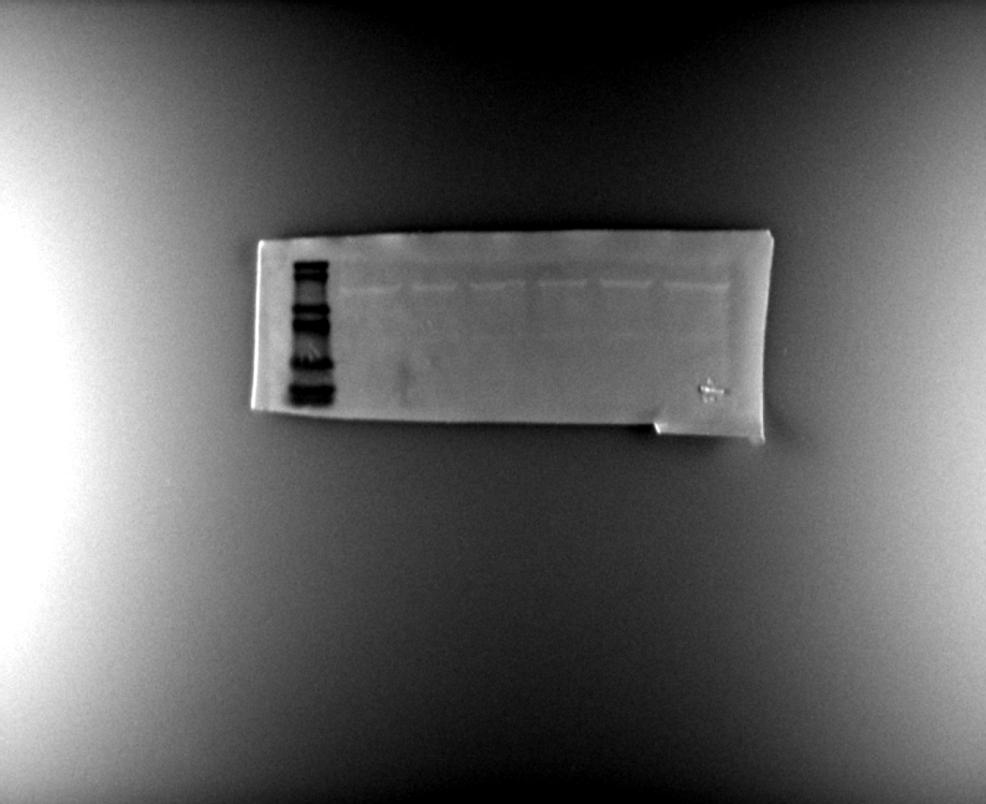
**

**73kDa**

**GCLC**

**-(3)-for Fig 2F**

**
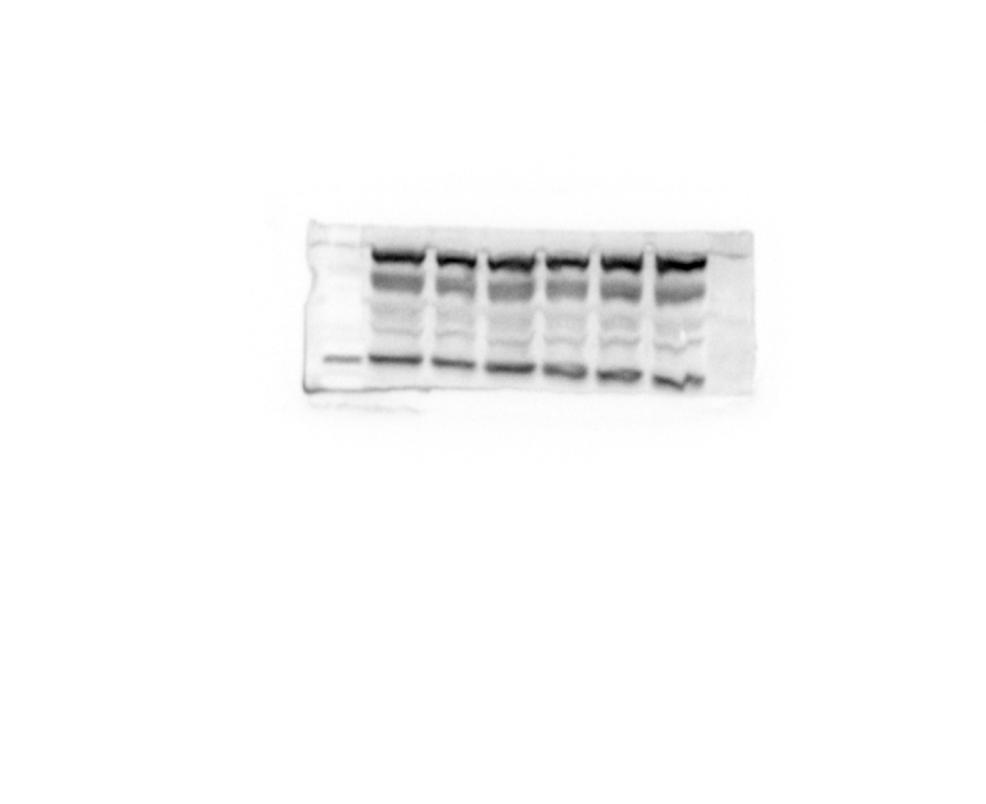

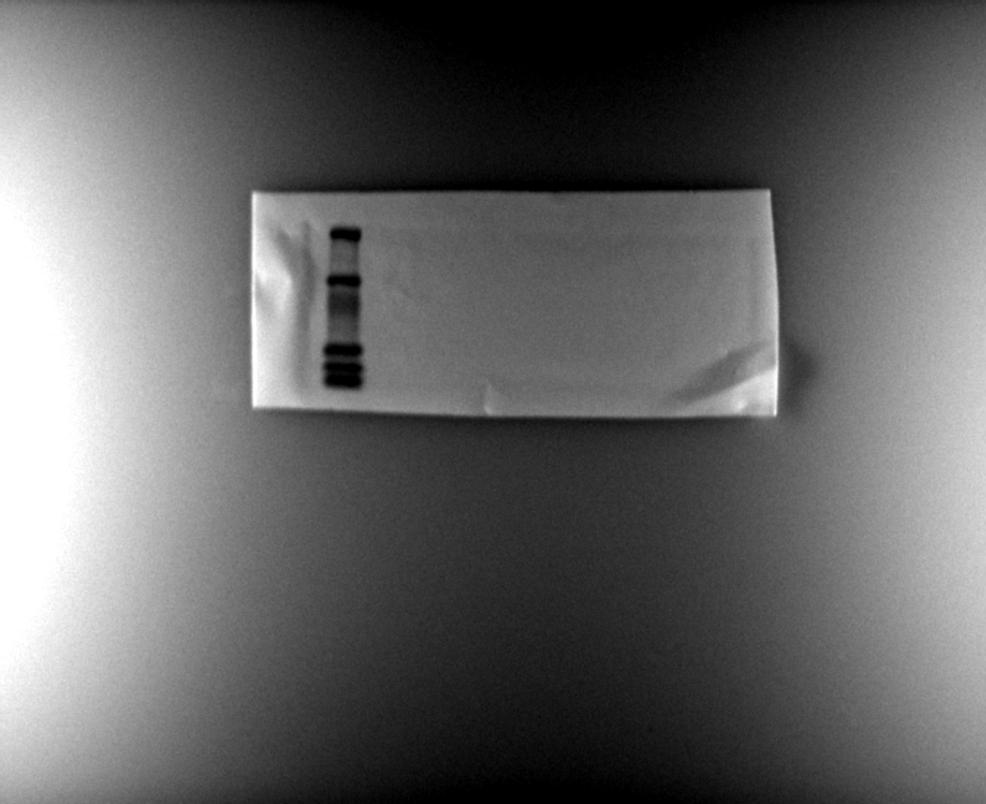
**

**73kDa**

**β-Actin**

**-(1)for Fig 2F**

**
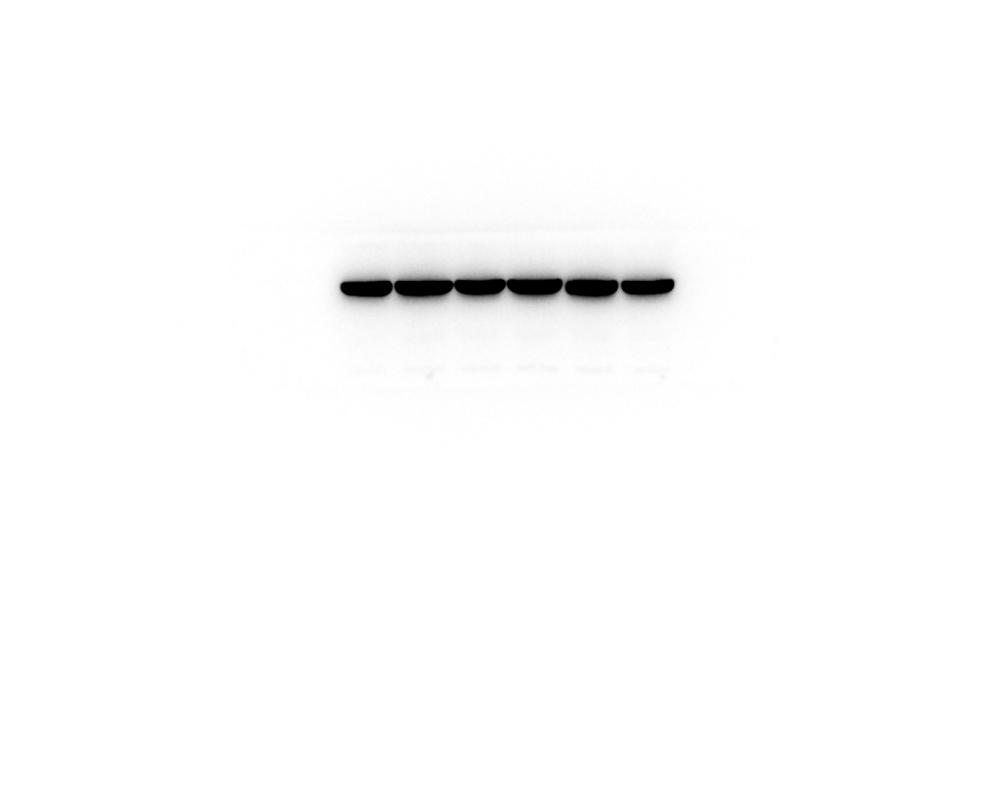

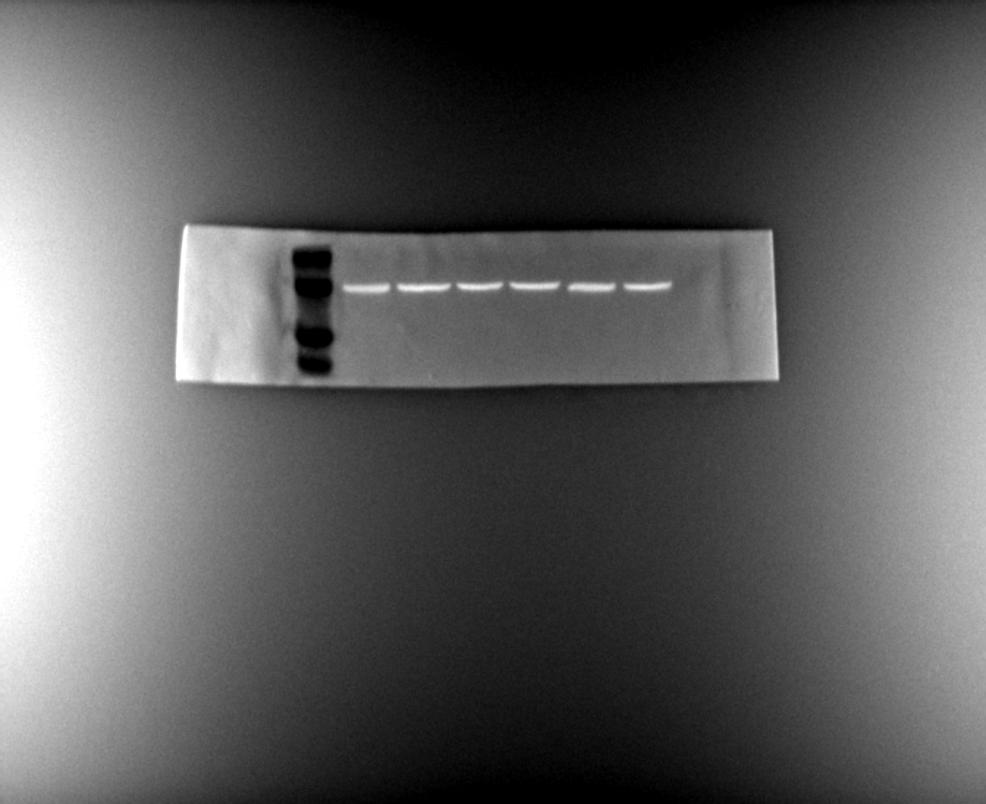
**

**42kDa**

**β-Actin**

**-(2)for Fig 2F**

**
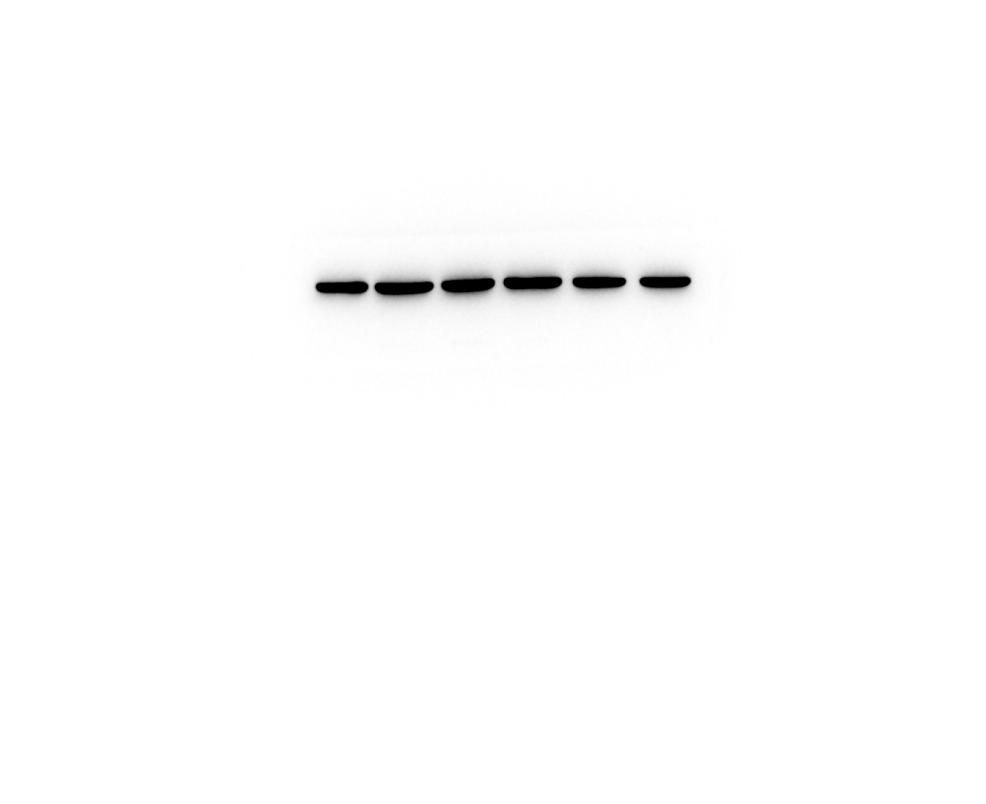
**

**42kDa**

**
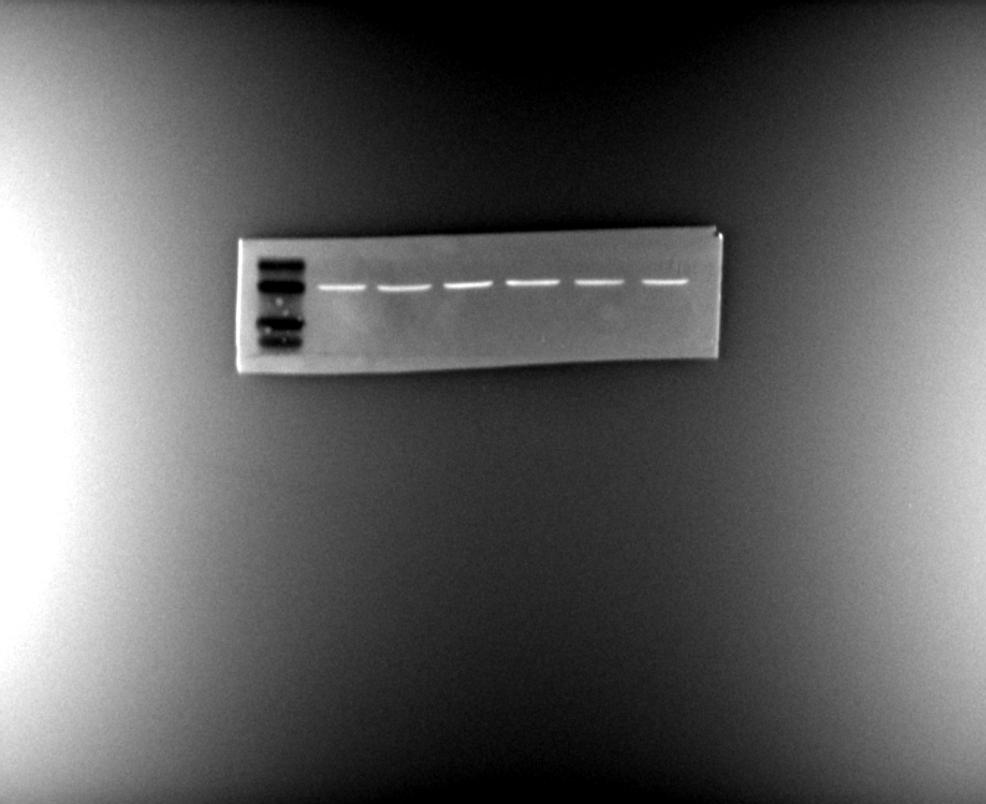
**

**β-Actin**

**-(3)for Fig 2F**

**
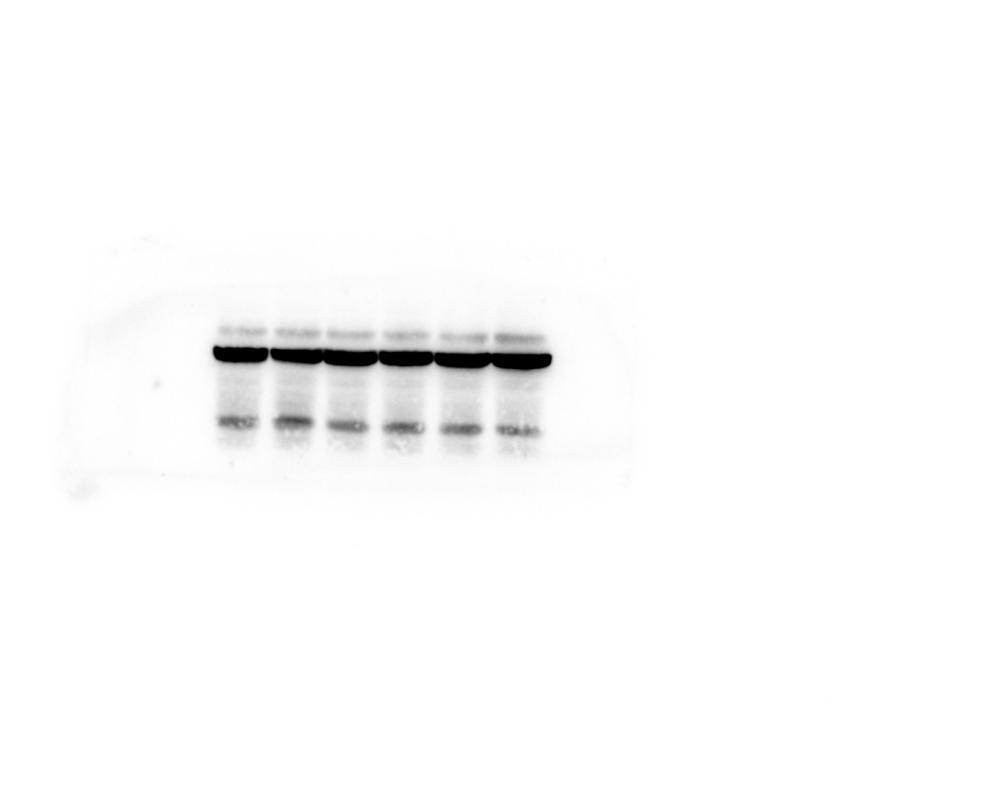
**

**42kDa**

**
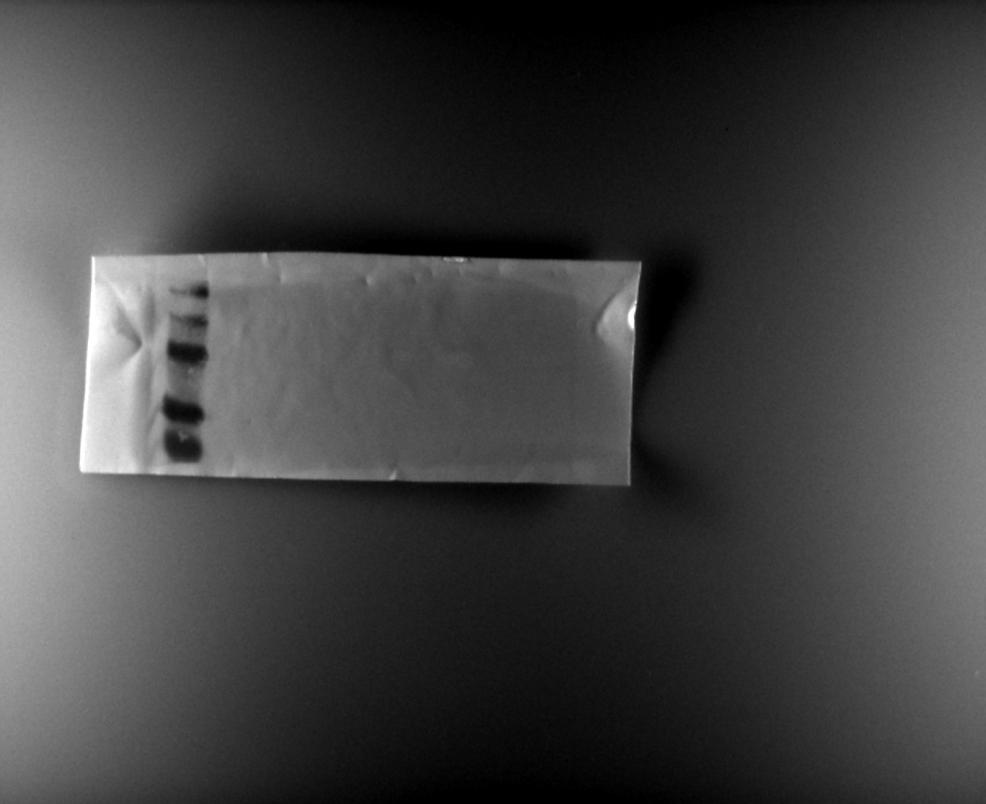
**
