# Supplementary figures and images for: The preventive effect of chlorogenic acid on cisplatin-induced acute kidney injury in mice
Source: Front Vet Sci. 2026 Feb 19;13:1763548. doi: 10.3389/fvets.2026.1763548 (PMC12960156; doi:10.3389/fvets.2026.1763548)

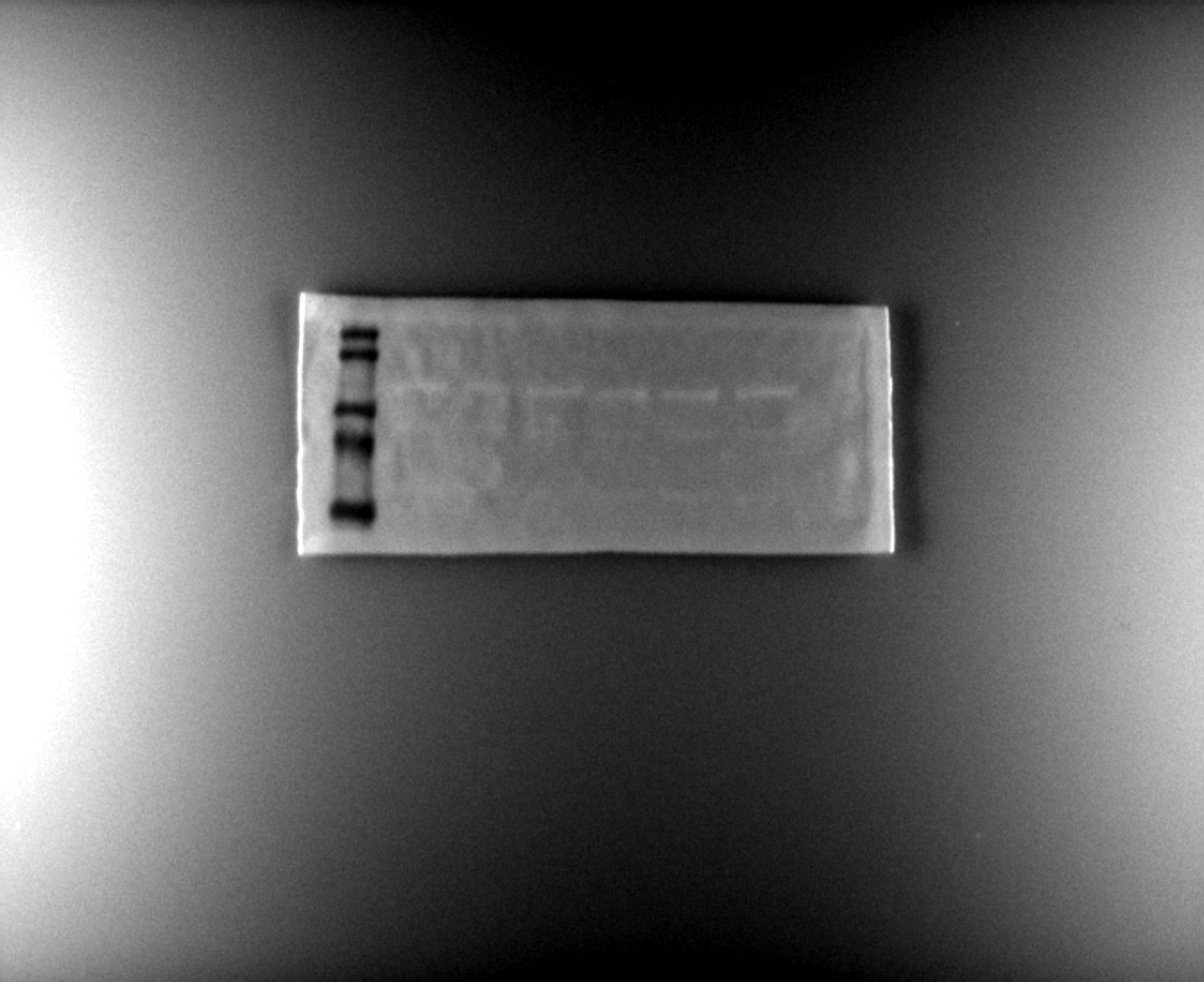

Supplement: Supplementary file 2 [file Data_Sheet_1.ZIP › RAW data MS ID1763548/Figure 2/Figure 2F WB images/GCLC/GCLC 1 in Fig 2F PVDF membrane.tif]

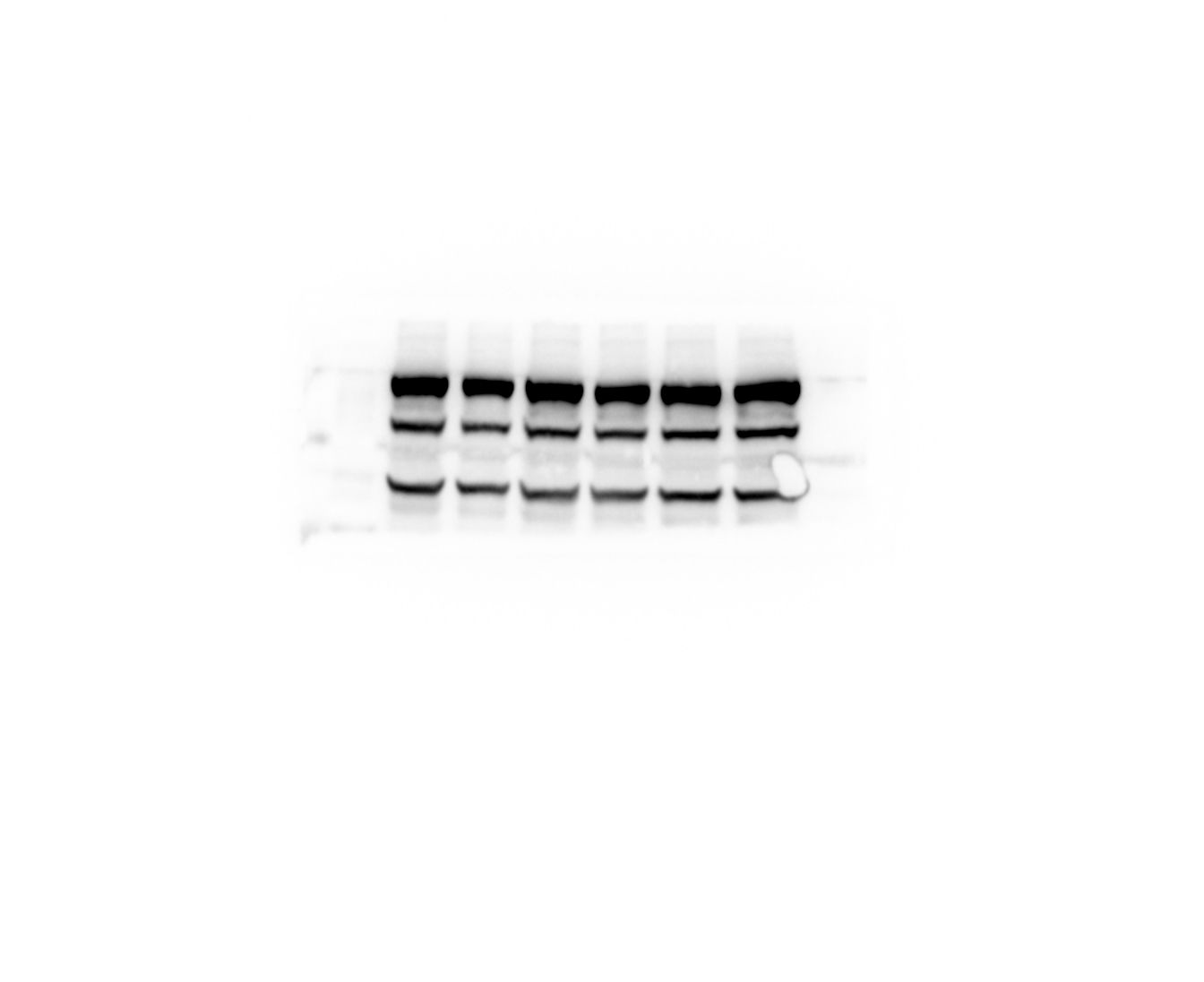

Supplement: Supplementary file 2 [file Data_Sheet_1.ZIP › RAW data MS ID1763548/Figure 2/Figure 2F WB images/GCLC/GCLC 1 in Fig 2F.tif]

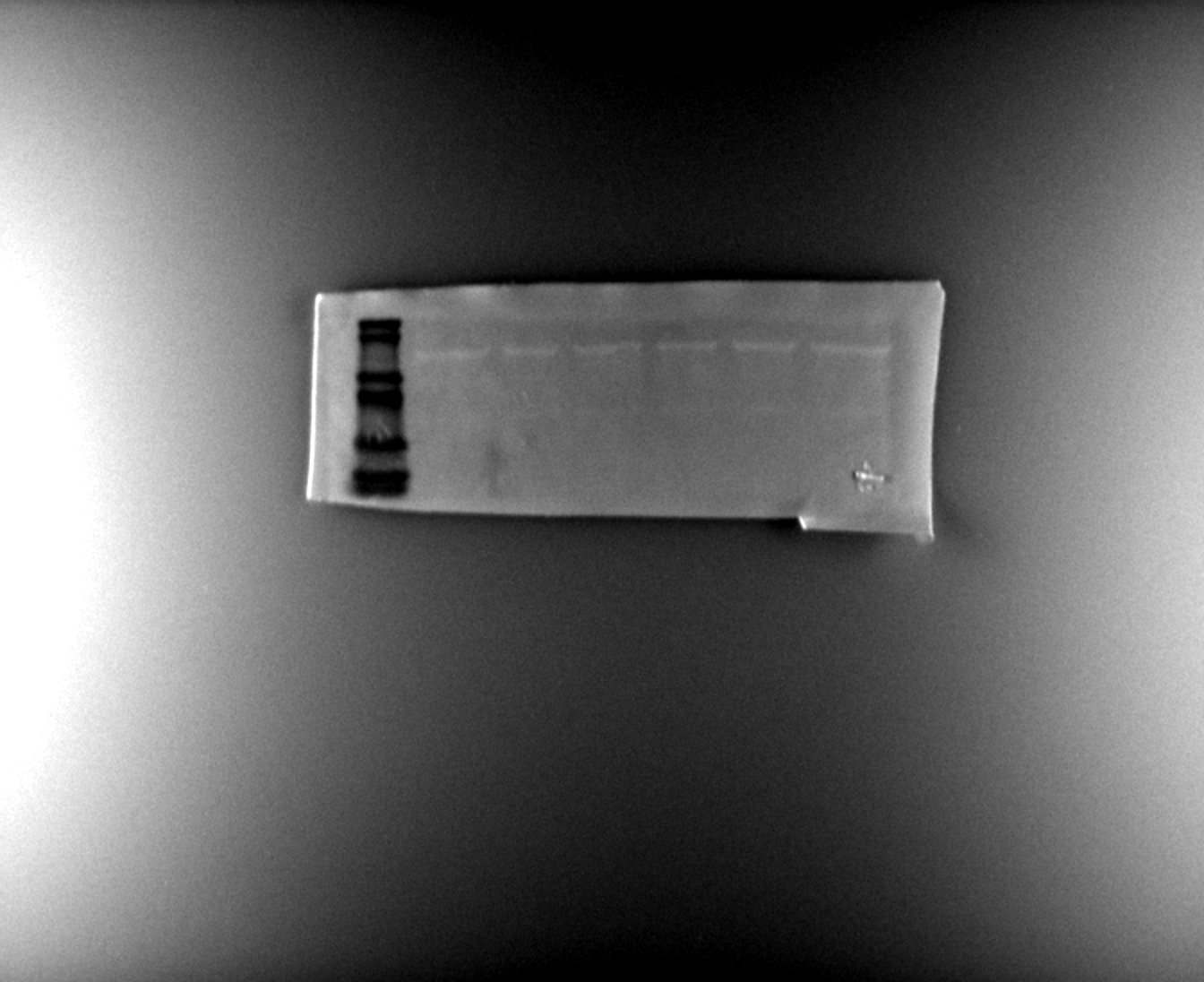

Supplement: Supplementary file 2 [file Data_Sheet_1.ZIP › RAW data MS ID1763548/Figure 2/Figure 2F WB images/GCLC/GCLC 2 PVDF membrane.tif]

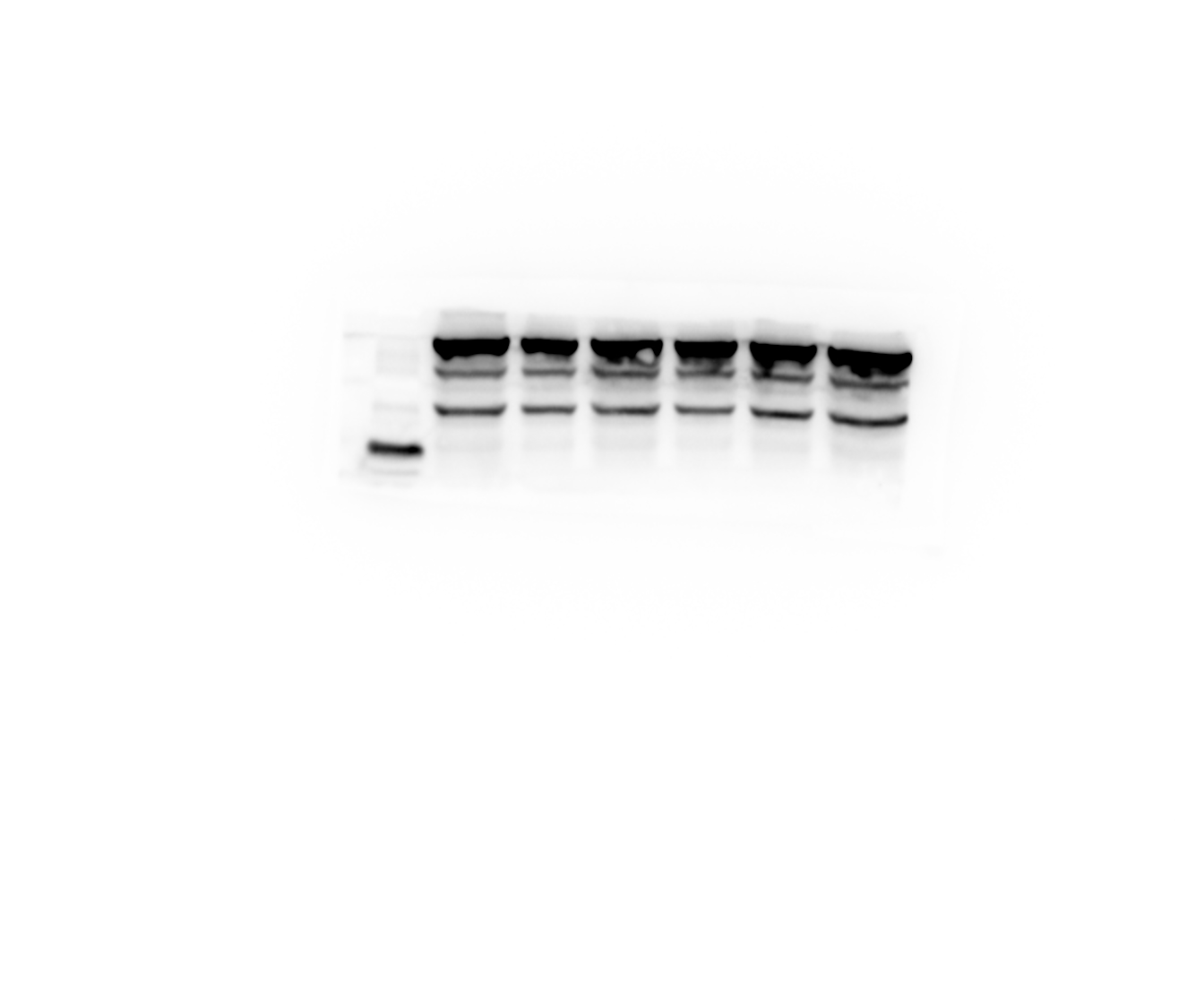

Supplement: Supplementary file 2 [file Data_Sheet_1.ZIP › RAW data MS ID1763548/Figure 2/Figure 2F WB images/GCLC/GCLC 2.tif]

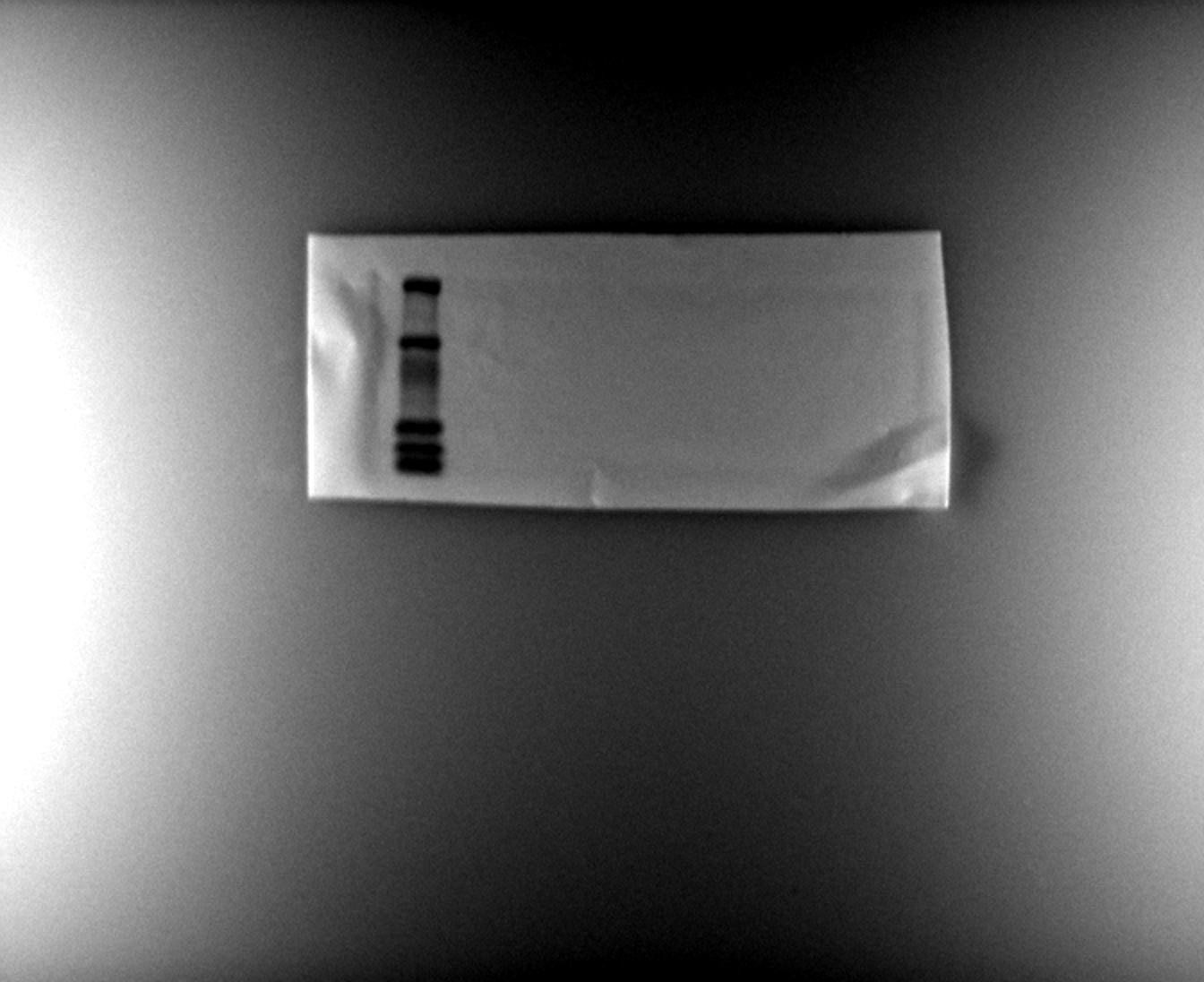

Supplement: Supplementary file 2 [file Data_Sheet_1.ZIP › RAW data MS ID1763548/Figure 2/Figure 2F WB images/GCLC/GCLC 3 PVDF membrane.tif]

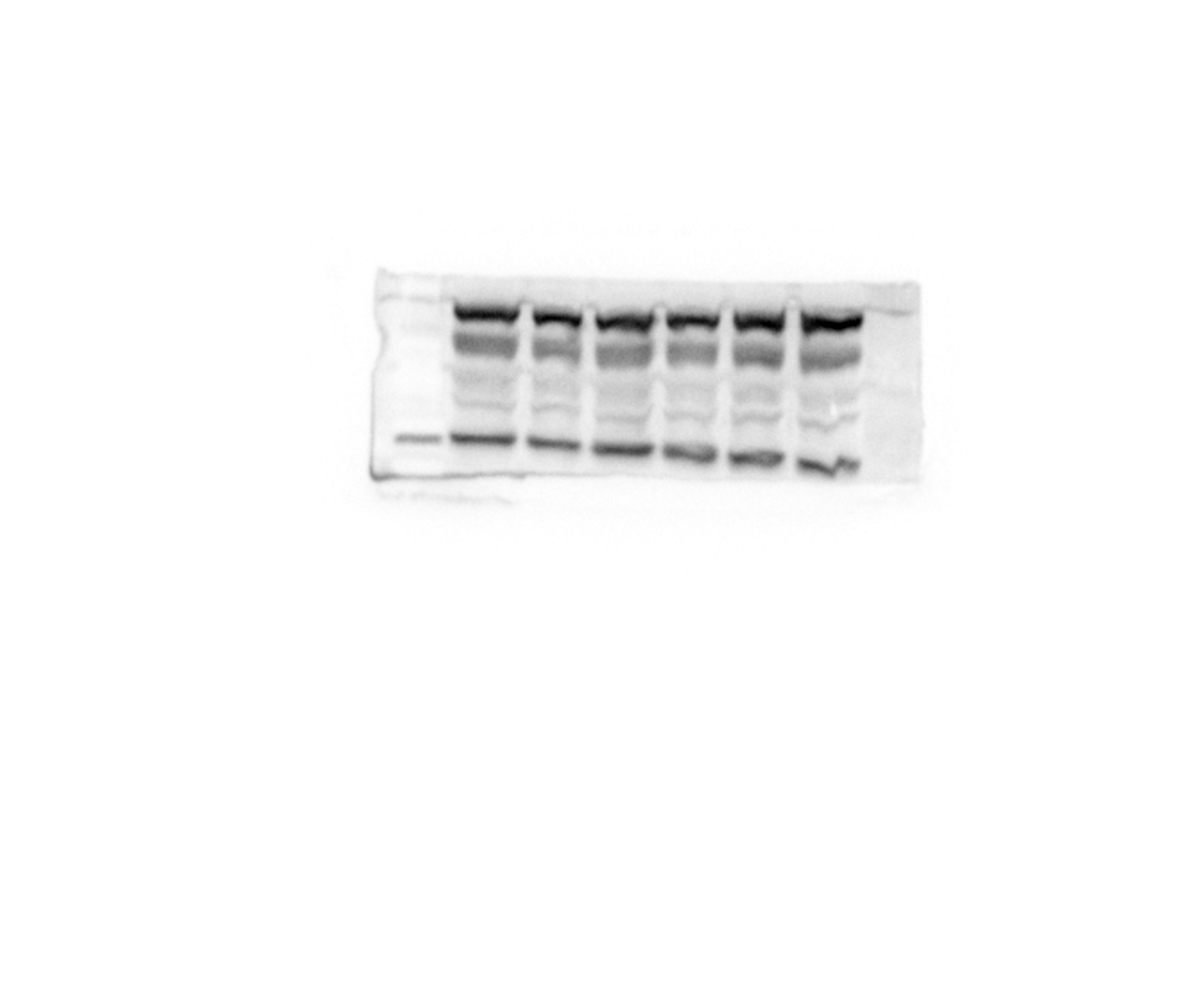

Supplement: Supplementary file 2 [file Data_Sheet_1.ZIP › RAW data MS ID1763548/Figure 2/Figure 2F WB images/GCLC/GCLC 3.tif]

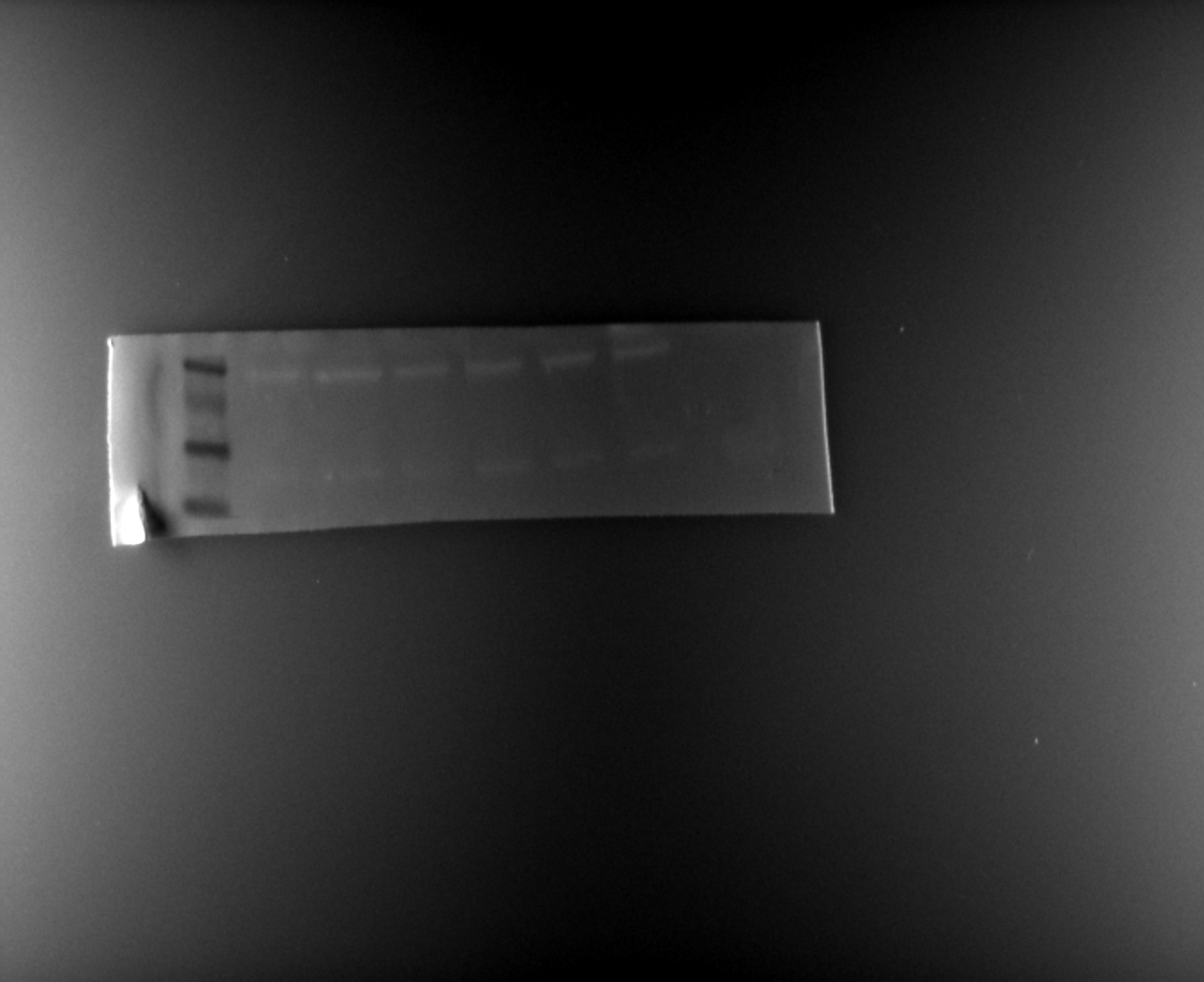

Supplement: Supplementary file 2 [file Data_Sheet_1.ZIP › RAW data MS ID1763548/Figure 2/Figure 2F WB images/Keap1/Keap1 1 in Fig 2F PVDF membrane.tif]

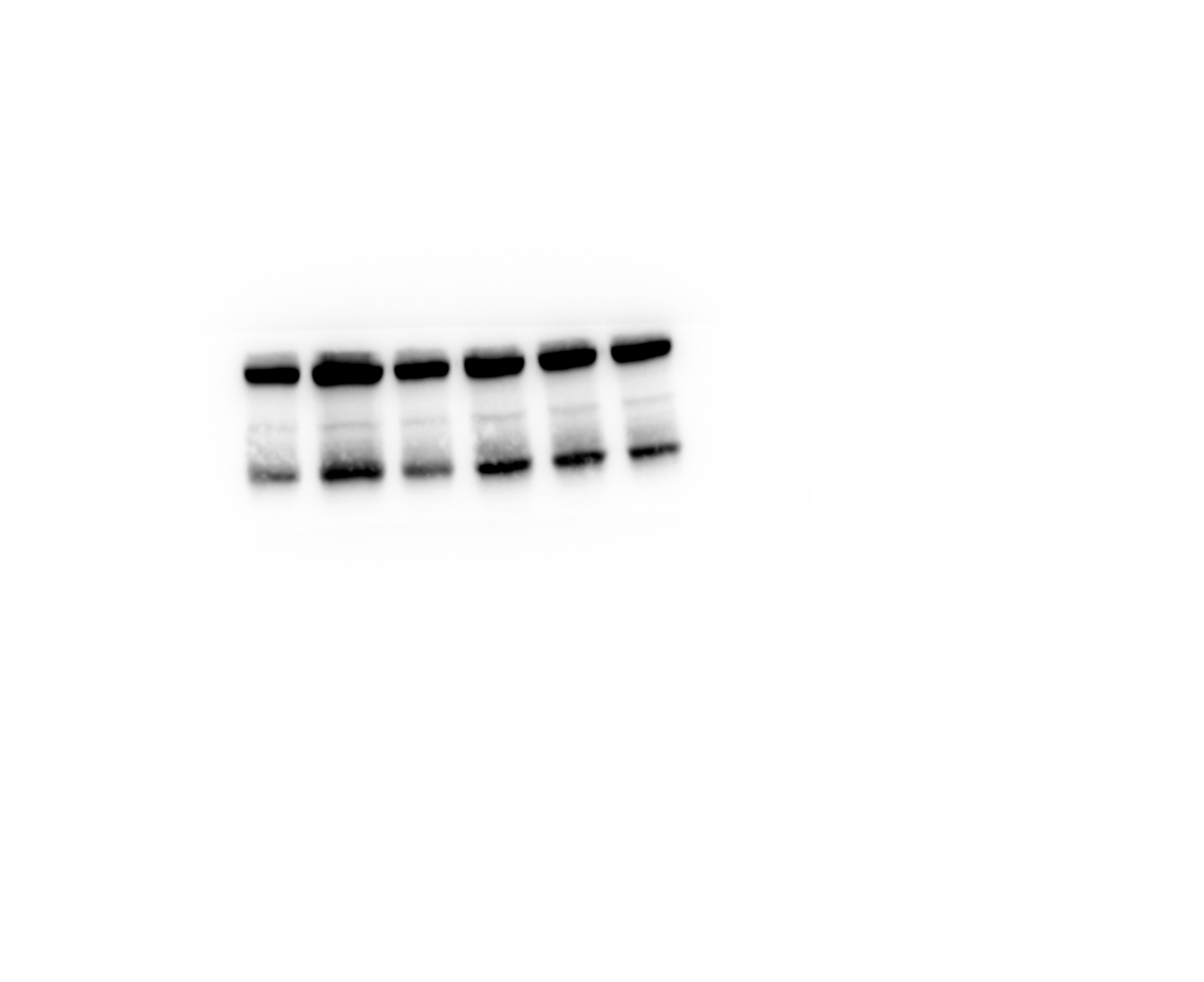

Supplement: Supplementary file 2 [file Data_Sheet_1.ZIP › RAW data MS ID1763548/Figure 2/Figure 2F WB images/Keap1/Keap1 1 in Fig 2F.tif]

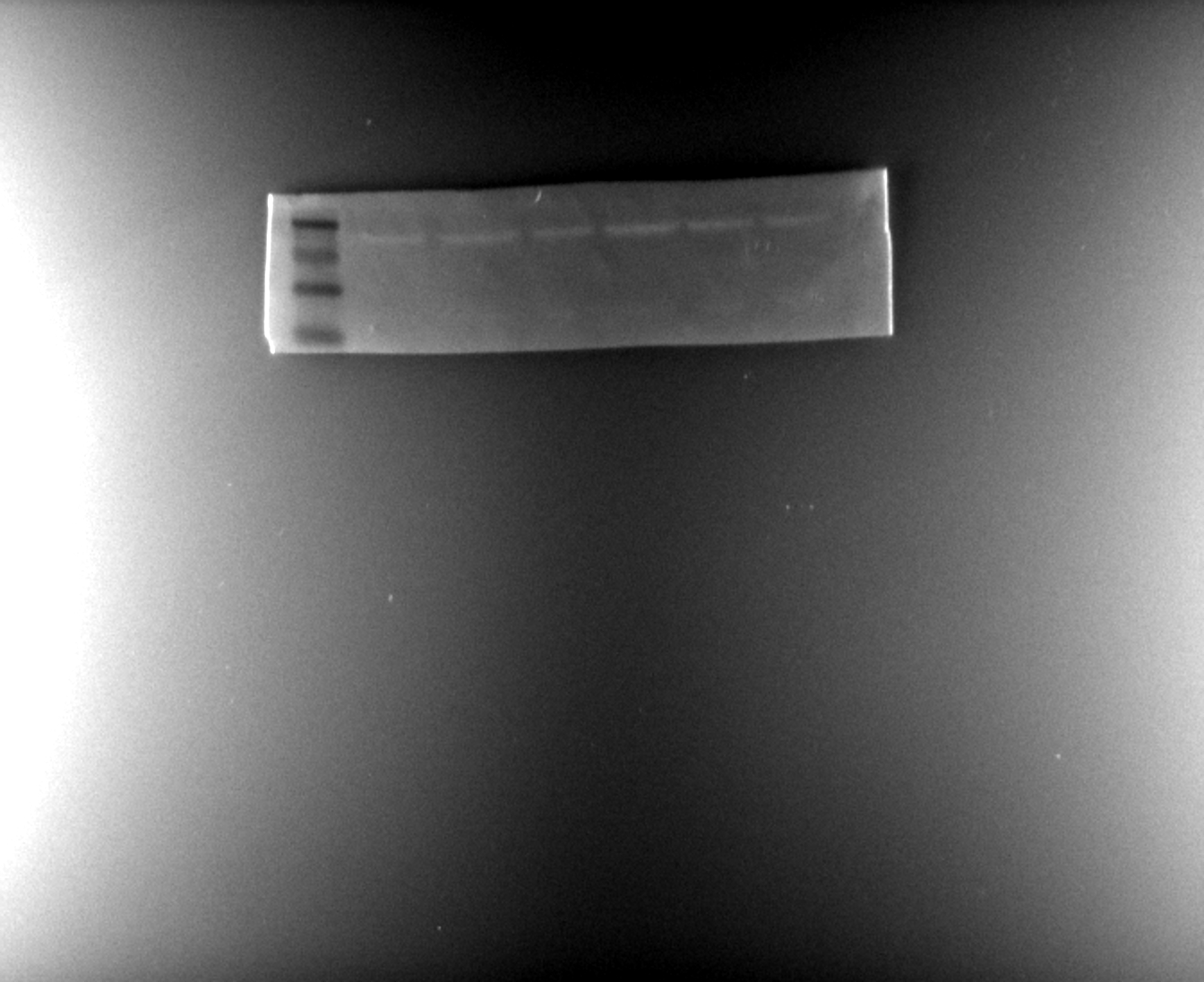

Supplement: Supplementary file 2 [file Data_Sheet_1.ZIP › RAW data MS ID1763548/Figure 2/Figure 2F WB images/Keap1/Keap1 2 PVDF membrane.tif]

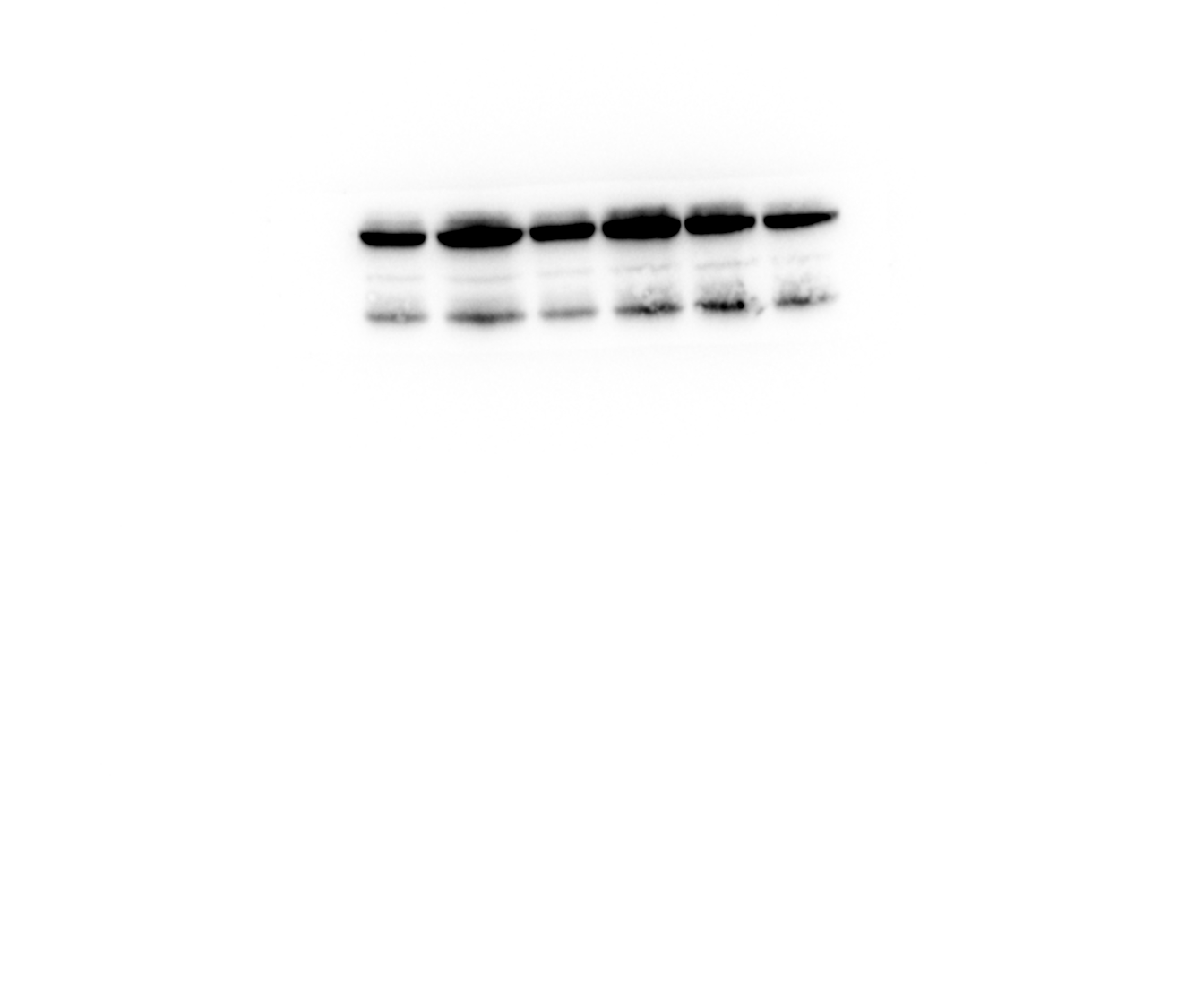

Supplement: Supplementary file 2 [file Data_Sheet_1.ZIP › RAW data MS ID1763548/Figure 2/Figure 2F WB images/Keap1/Keap1 2.tif]

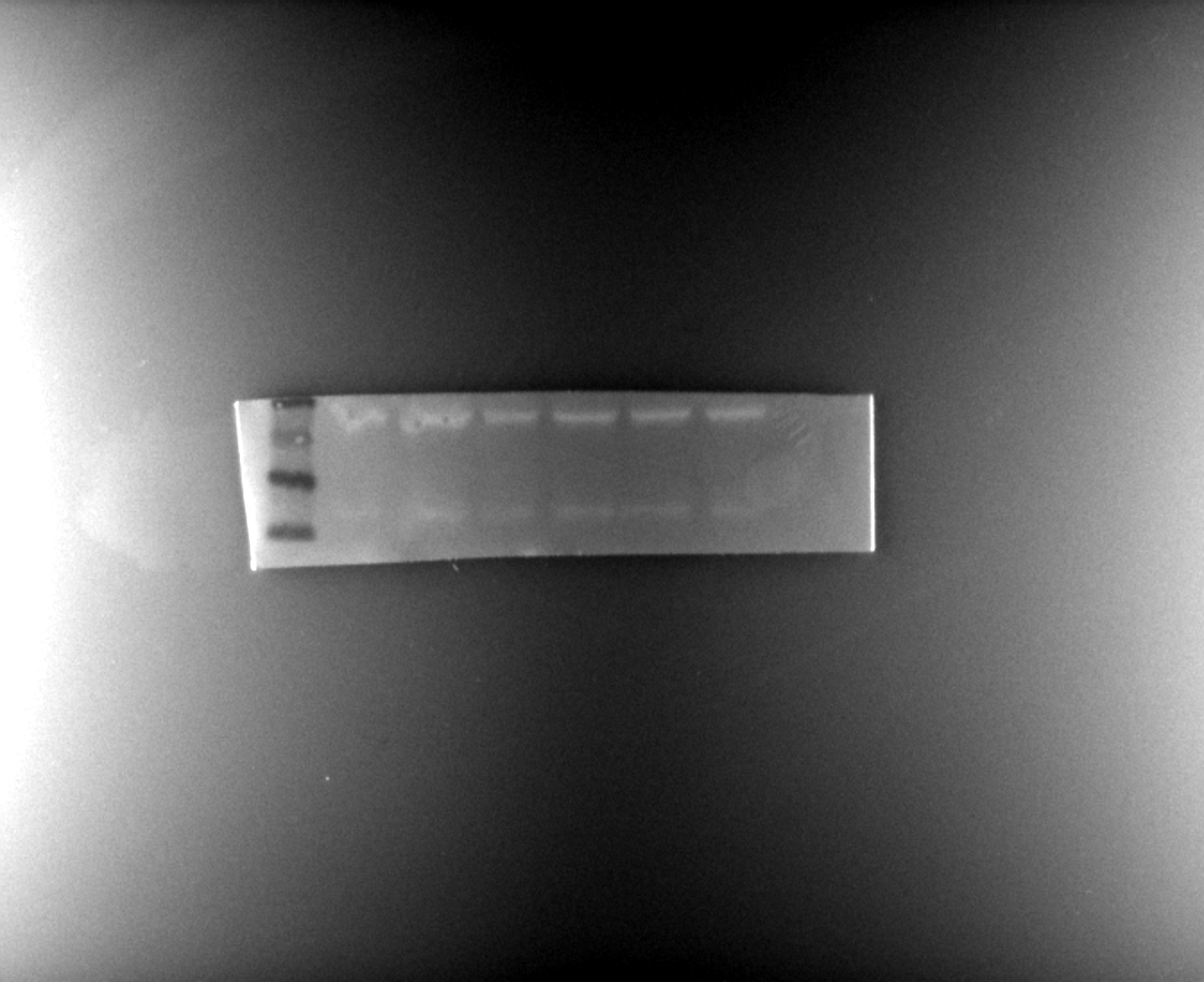

Supplement: Supplementary file 2 [file Data_Sheet_1.ZIP › RAW data MS ID1763548/Figure 2/Figure 2F WB images/Keap1/Keap1 3 PVDF membrane.tif]

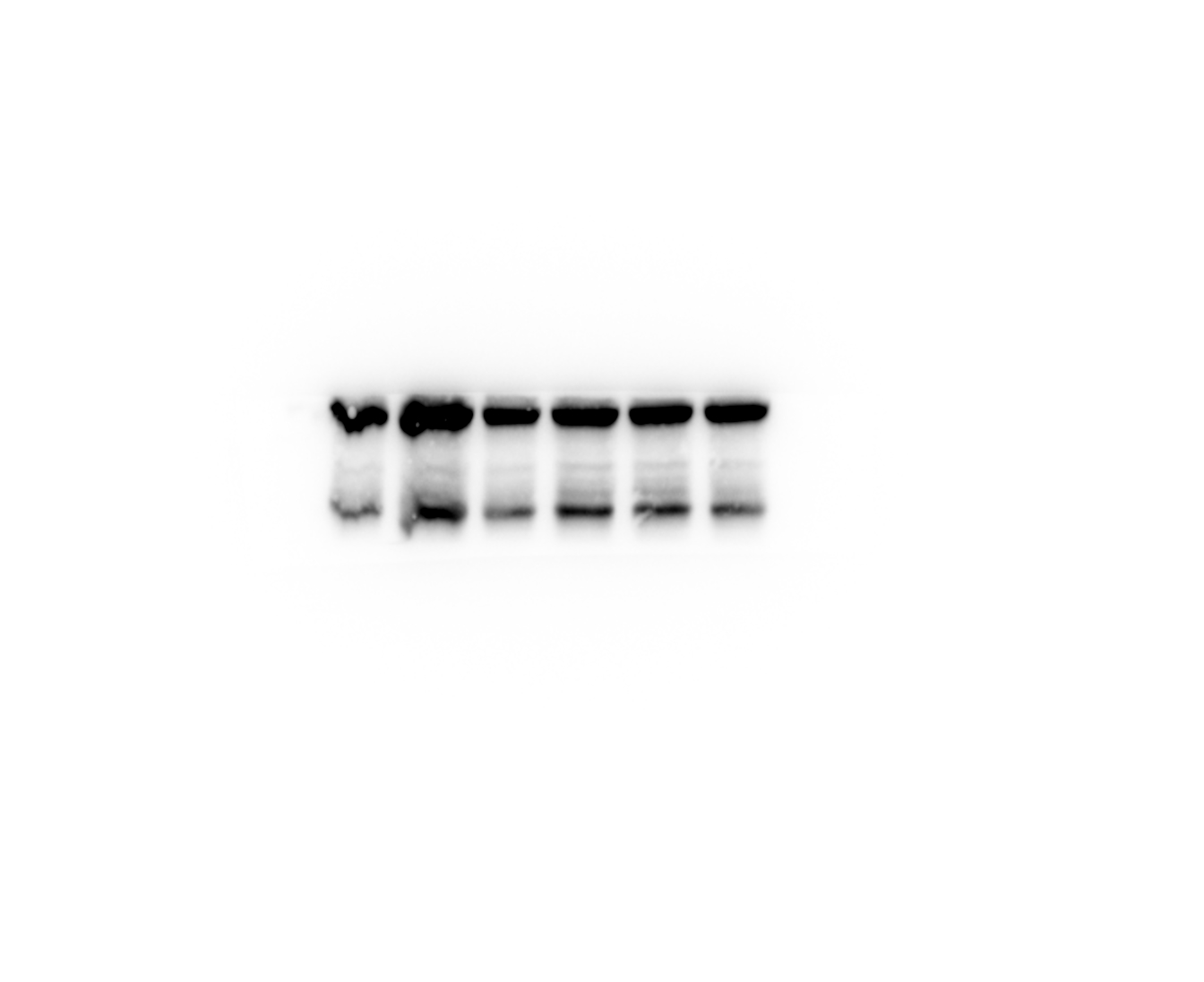

Supplement: Supplementary file 2 [file Data_Sheet_1.ZIP › RAW data MS ID1763548/Figure 2/Figure 2F WB images/Keap1/Keap1 3.tif]

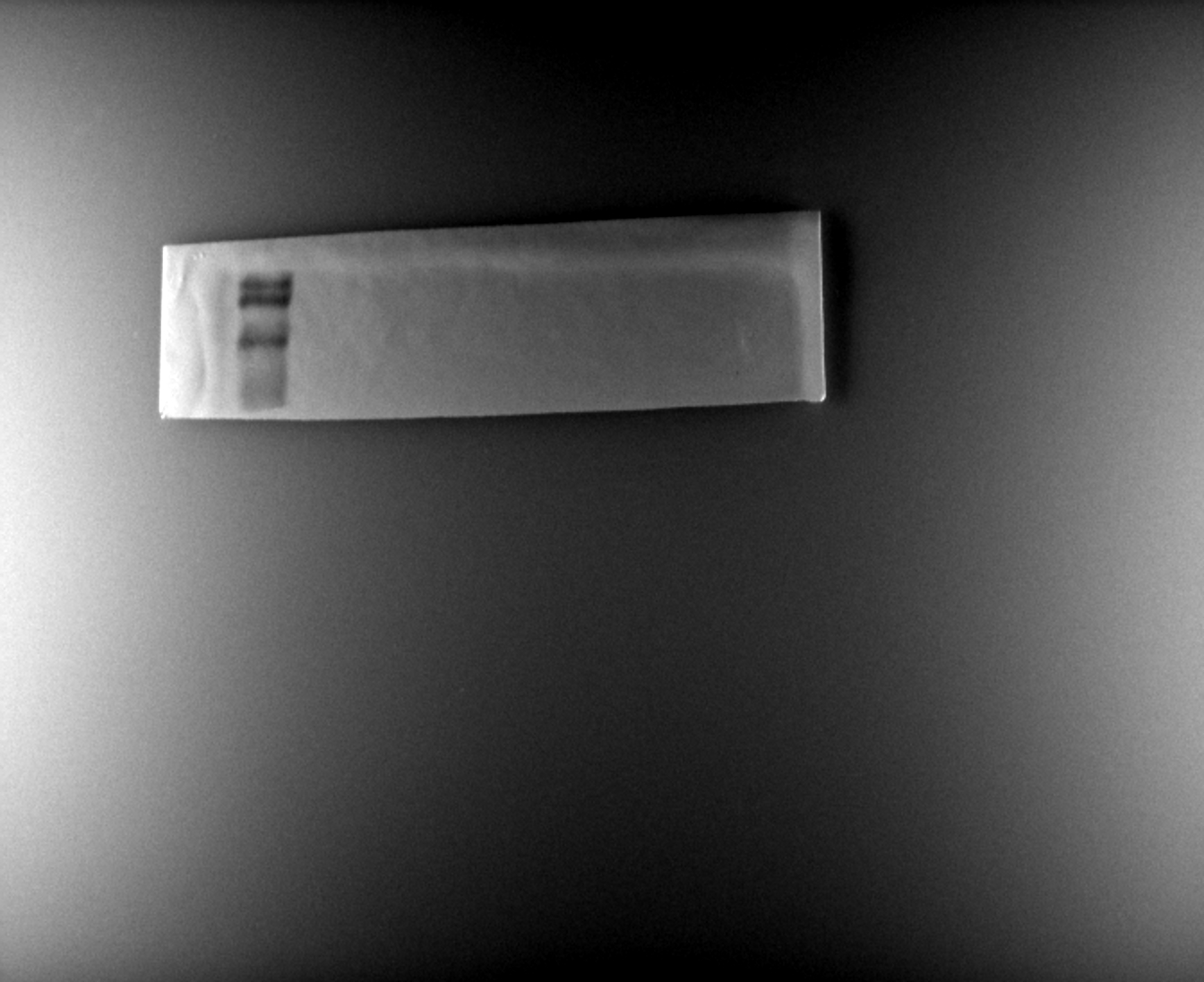

Supplement: Supplementary file 2 [file Data_Sheet_1.ZIP › RAW data MS ID1763548/Figure 2/Figure 2F WB images/Nrf2/Nrf2 1 in Fig 2F PVDF membrane.tif]

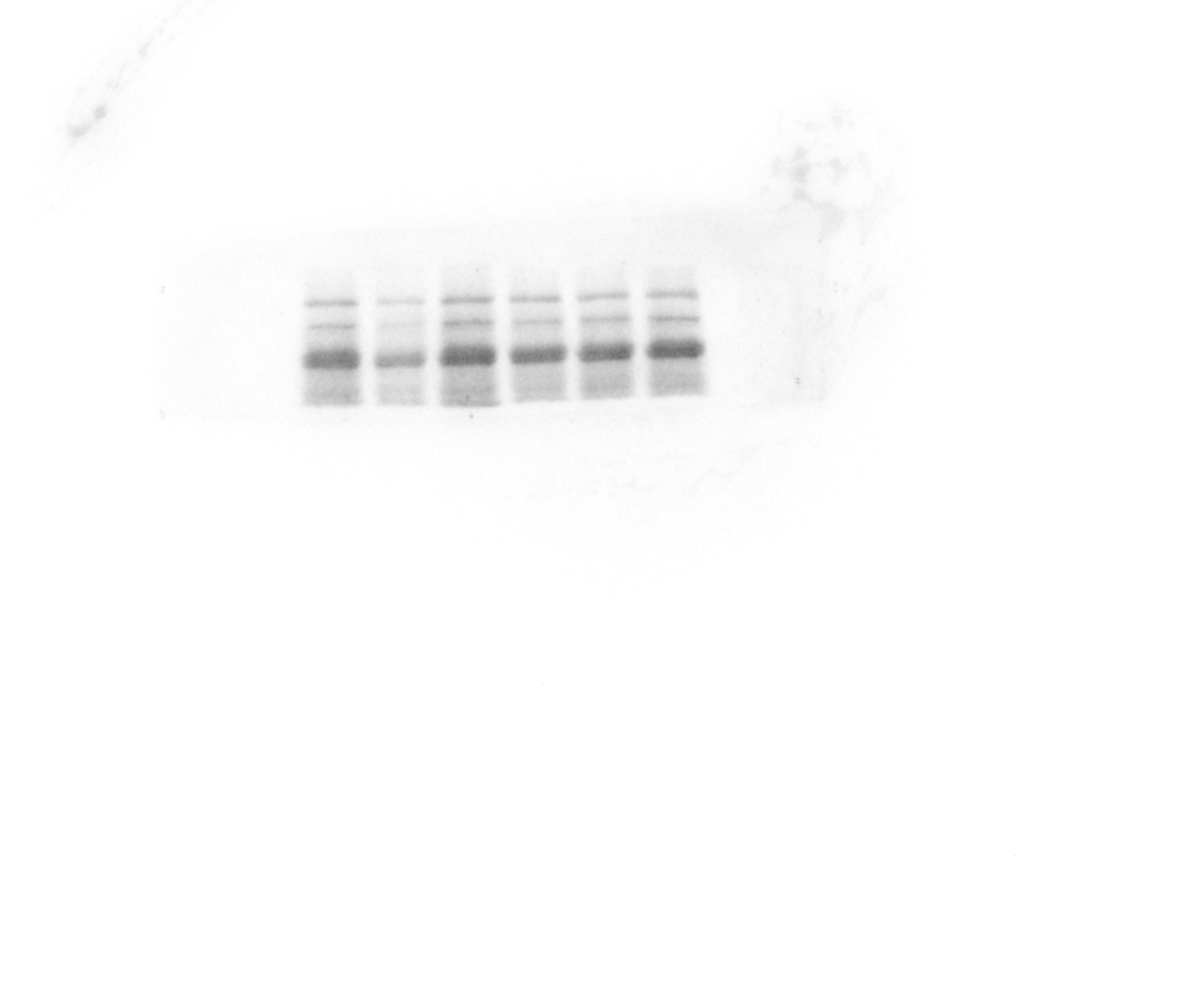

Supplement: Supplementary file 2 [file Data_Sheet_1.ZIP › RAW data MS ID1763548/Figure 2/Figure 2F WB images/Nrf2/Nrf2 1 in Fig 2F.tif]

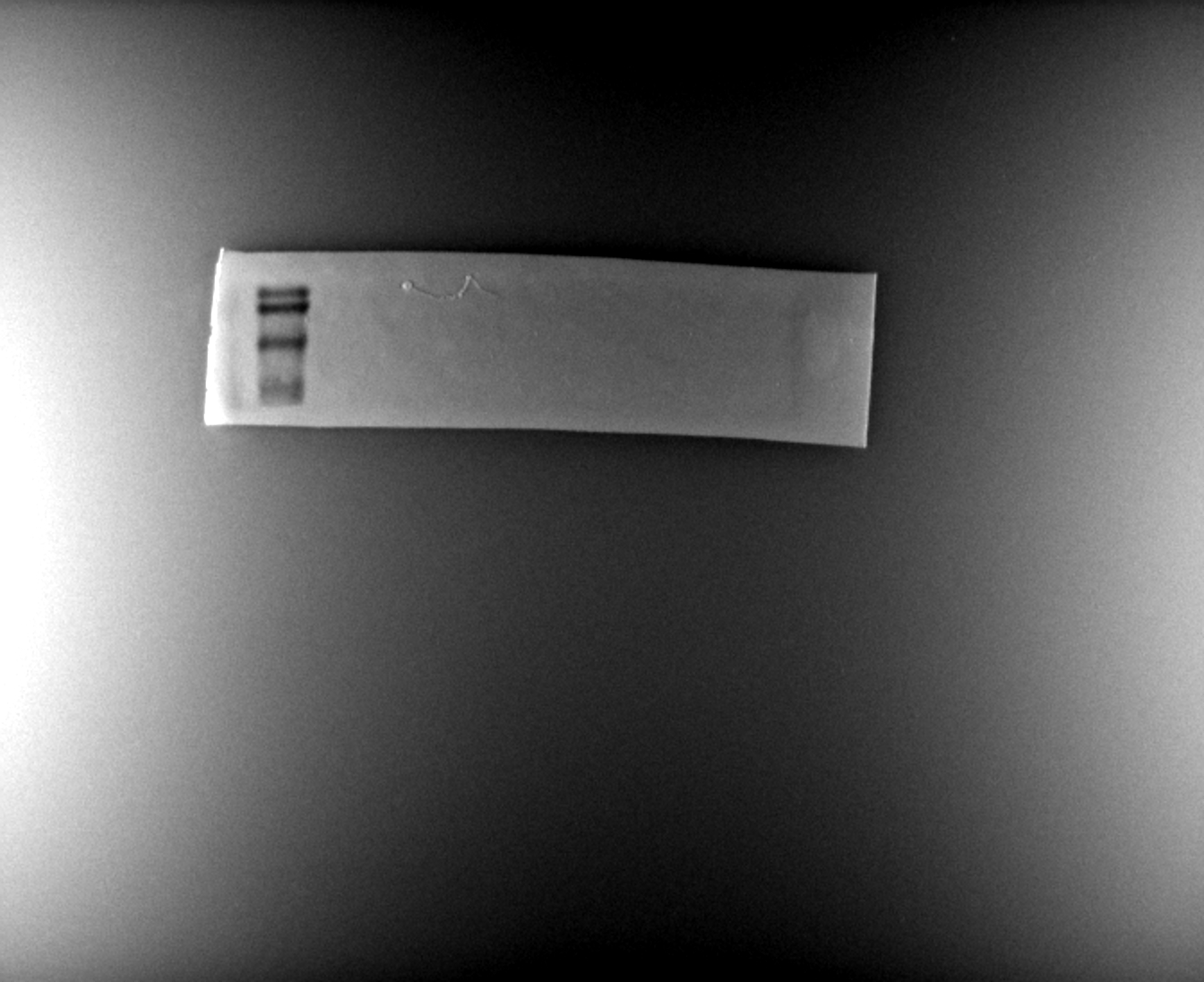

Supplement: Supplementary file 2 [file Data_Sheet_1.ZIP › RAW data MS ID1763548/Figure 2/Figure 2F WB images/Nrf2/Nrf2 2 PVDF membrane.tif]

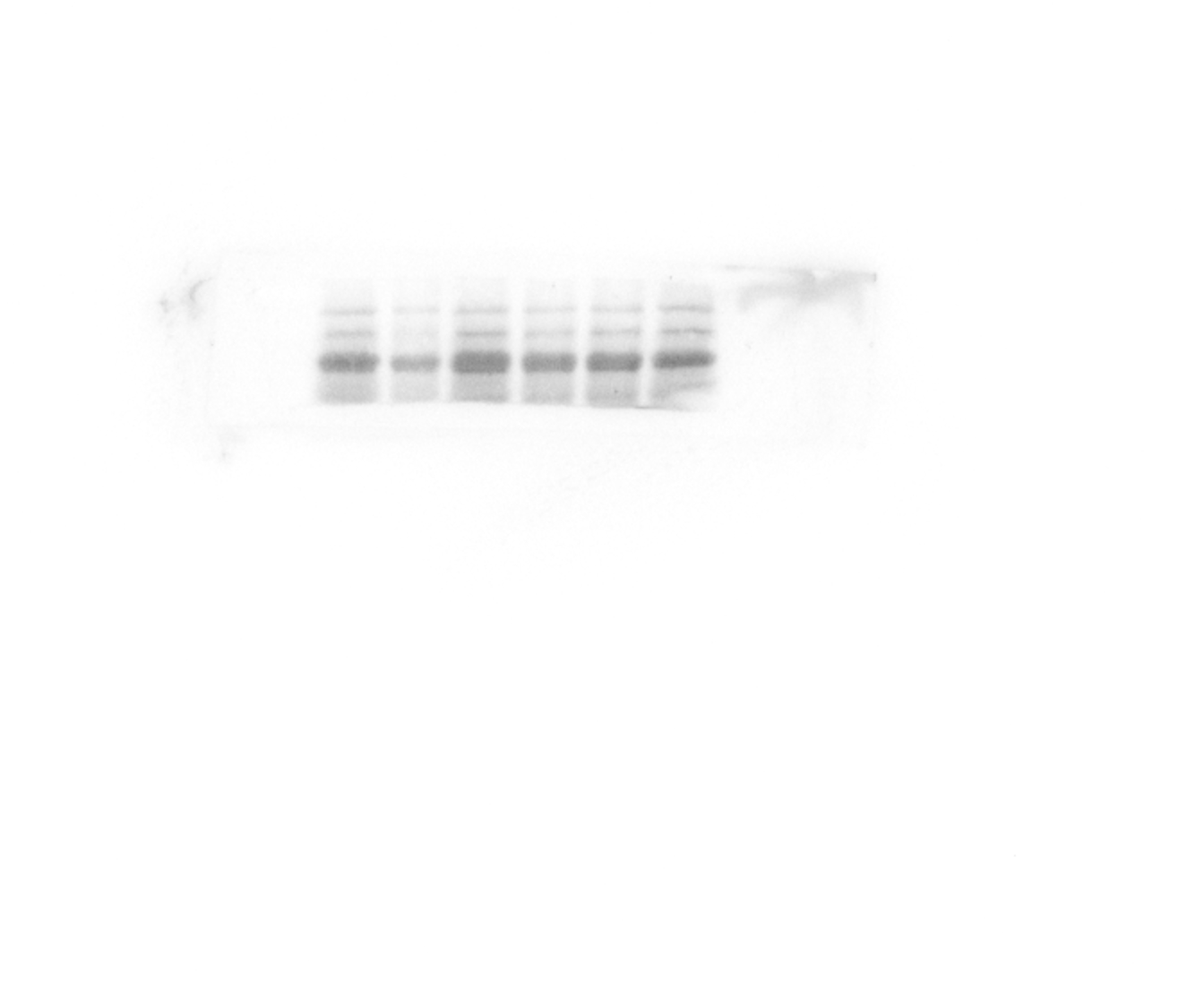

Supplement: Supplementary file 2 [file Data_Sheet_1.ZIP › RAW data MS ID1763548/Figure 2/Figure 2F WB images/Nrf2/Nrf2 2.tif]

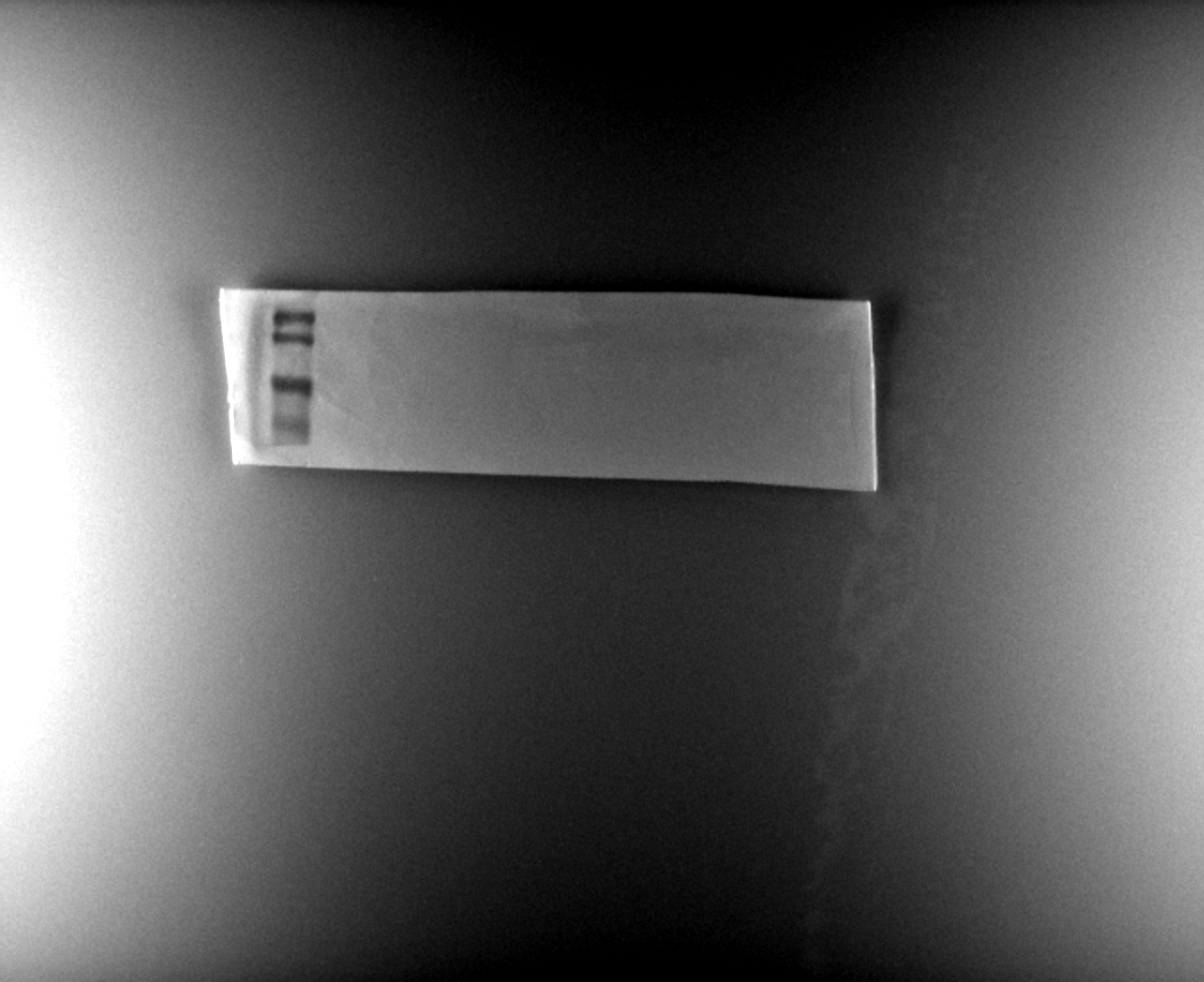

Supplement: Supplementary file 2 [file Data_Sheet_1.ZIP › RAW data MS ID1763548/Figure 2/Figure 2F WB images/Nrf2/Nrf2 3 PVDF membrane.tif]

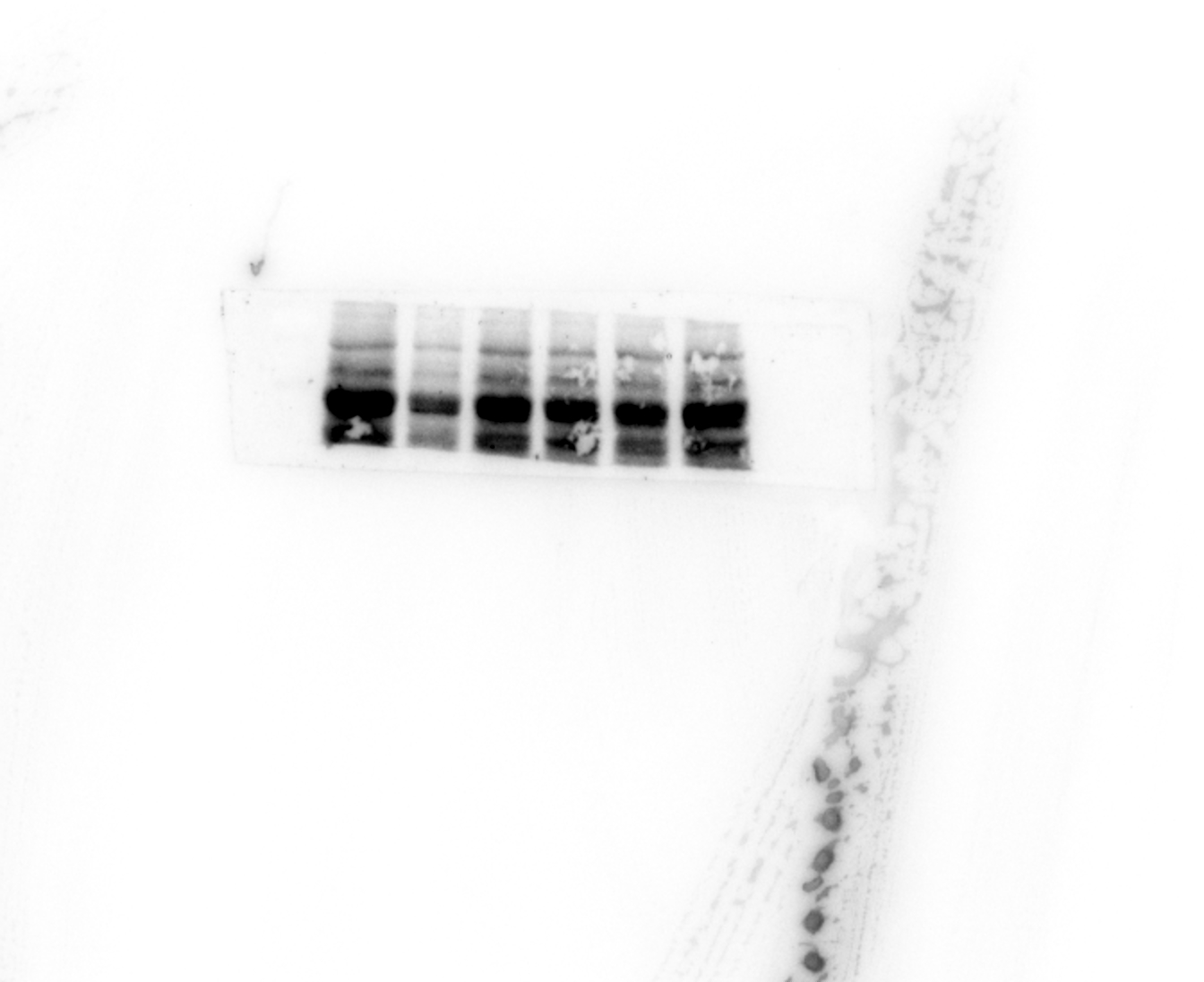

Supplement: Supplementary file 2 [file Data_Sheet_1.ZIP › RAW data MS ID1763548/Figure 2/Figure 2F WB images/Nrf2/Nrf2 3.tif]

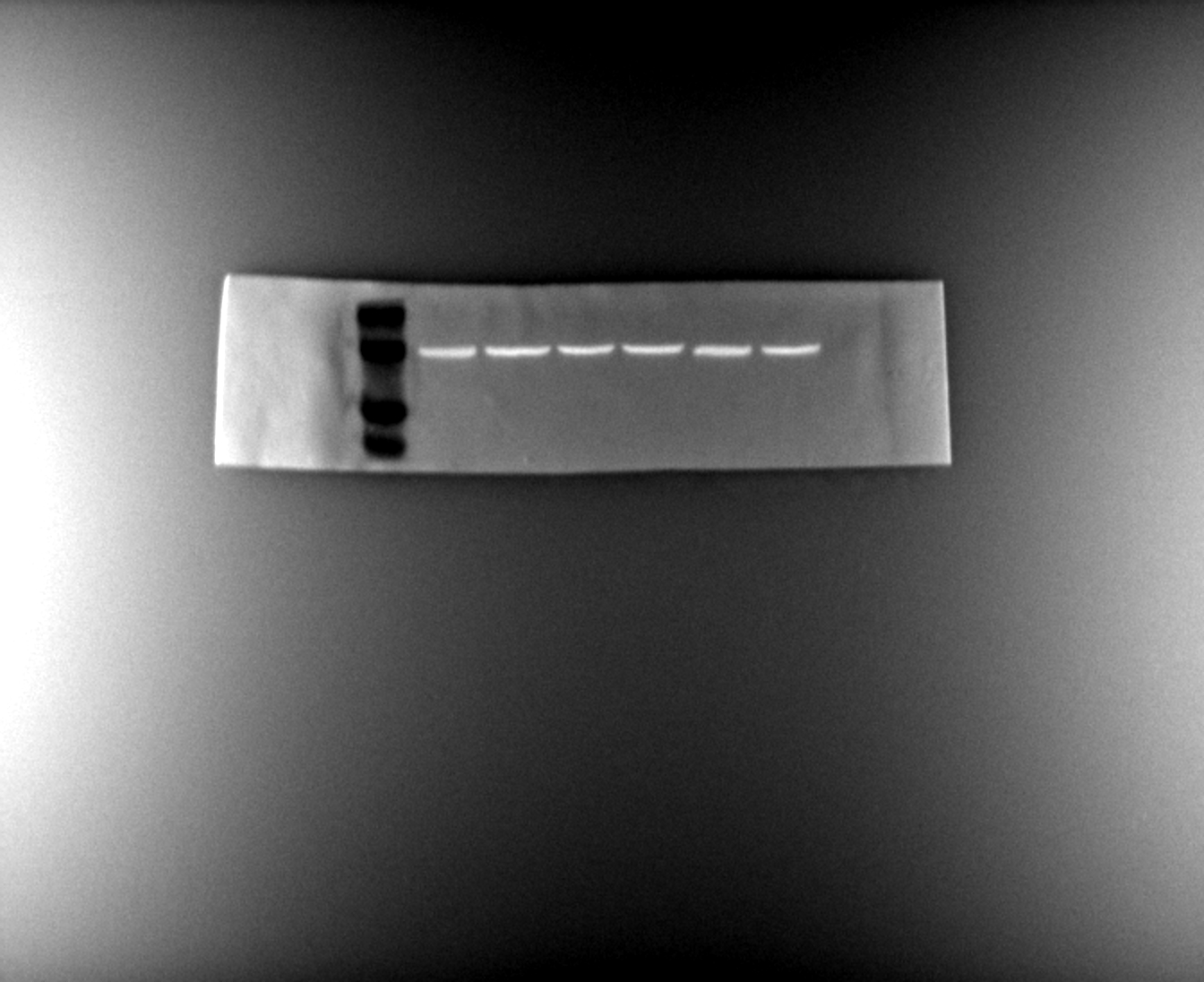

Supplement: Supplementary file 2 [file Data_Sheet_1.ZIP › RAW data MS ID1763548/Figure 2/Figure 2F WB images/a┬-actin/a┬-actin 1 in Fig 2F PVDF membrane.tif]

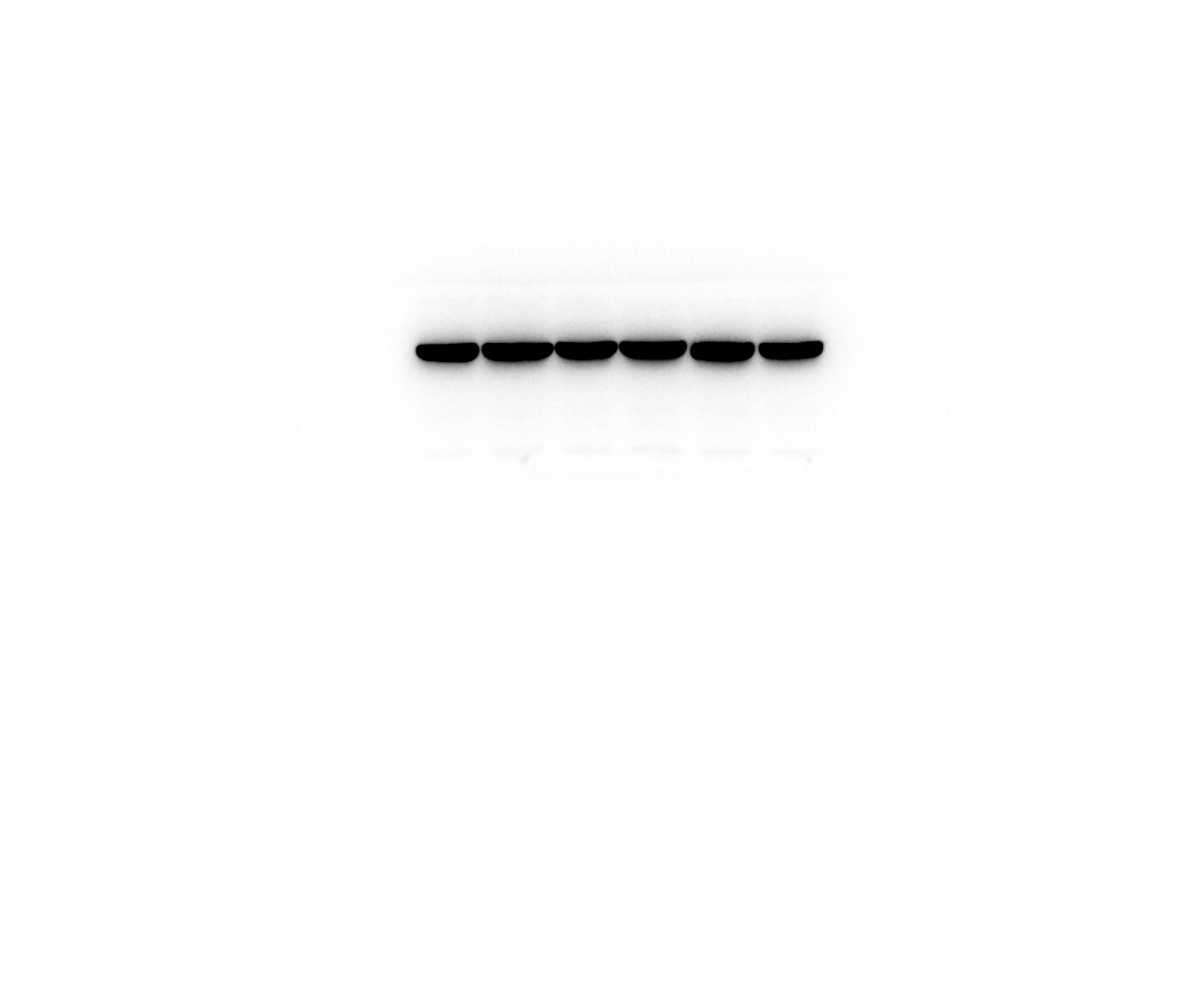

Supplement: Supplementary file 2 [file Data_Sheet_1.ZIP › RAW data MS ID1763548/Figure 2/Figure 2F WB images/a┬-actin/a┬-actin 1 in Fig 2F.tif]

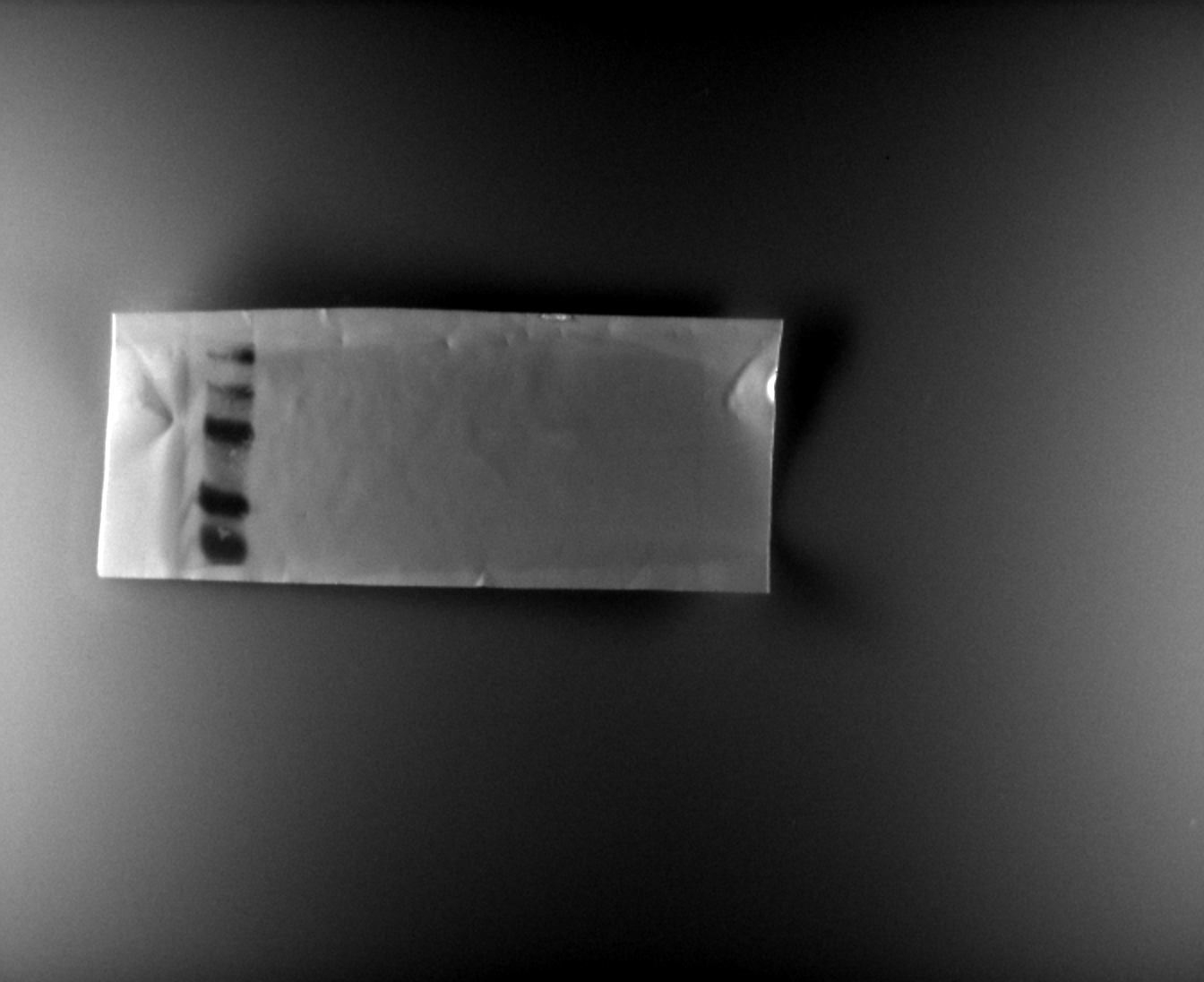

Supplement: Supplementary file 2 [file Data_Sheet_1.ZIP › RAW data MS ID1763548/Figure 2/Figure 2F WB images/a┬-actin/a┬-actin 2 PVDF membrane.tif]

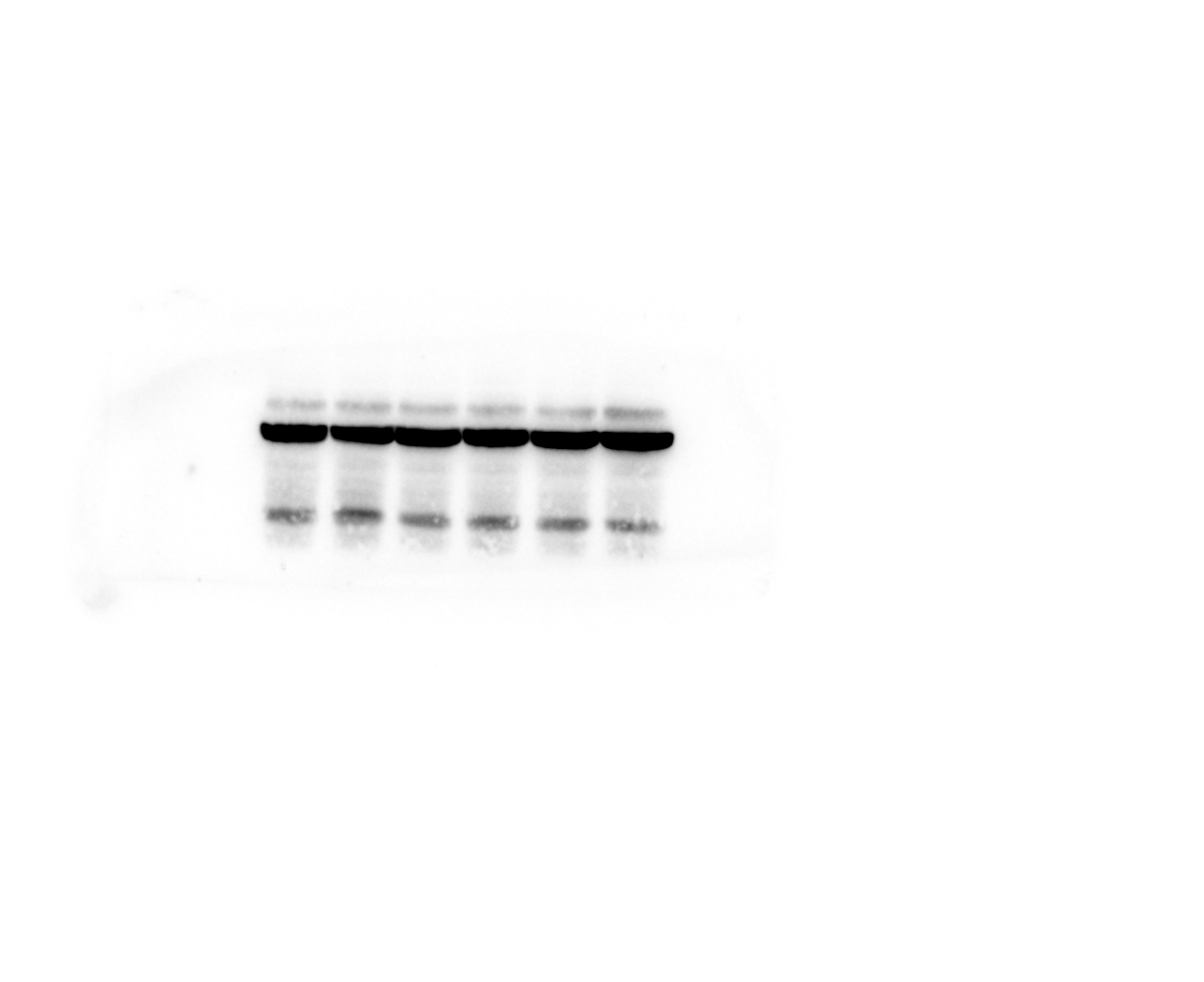

Supplement: Supplementary file 2 [file Data_Sheet_1.ZIP › RAW data MS ID1763548/Figure 2/Figure 2F WB images/a┬-actin/a┬-actin 2.tif]

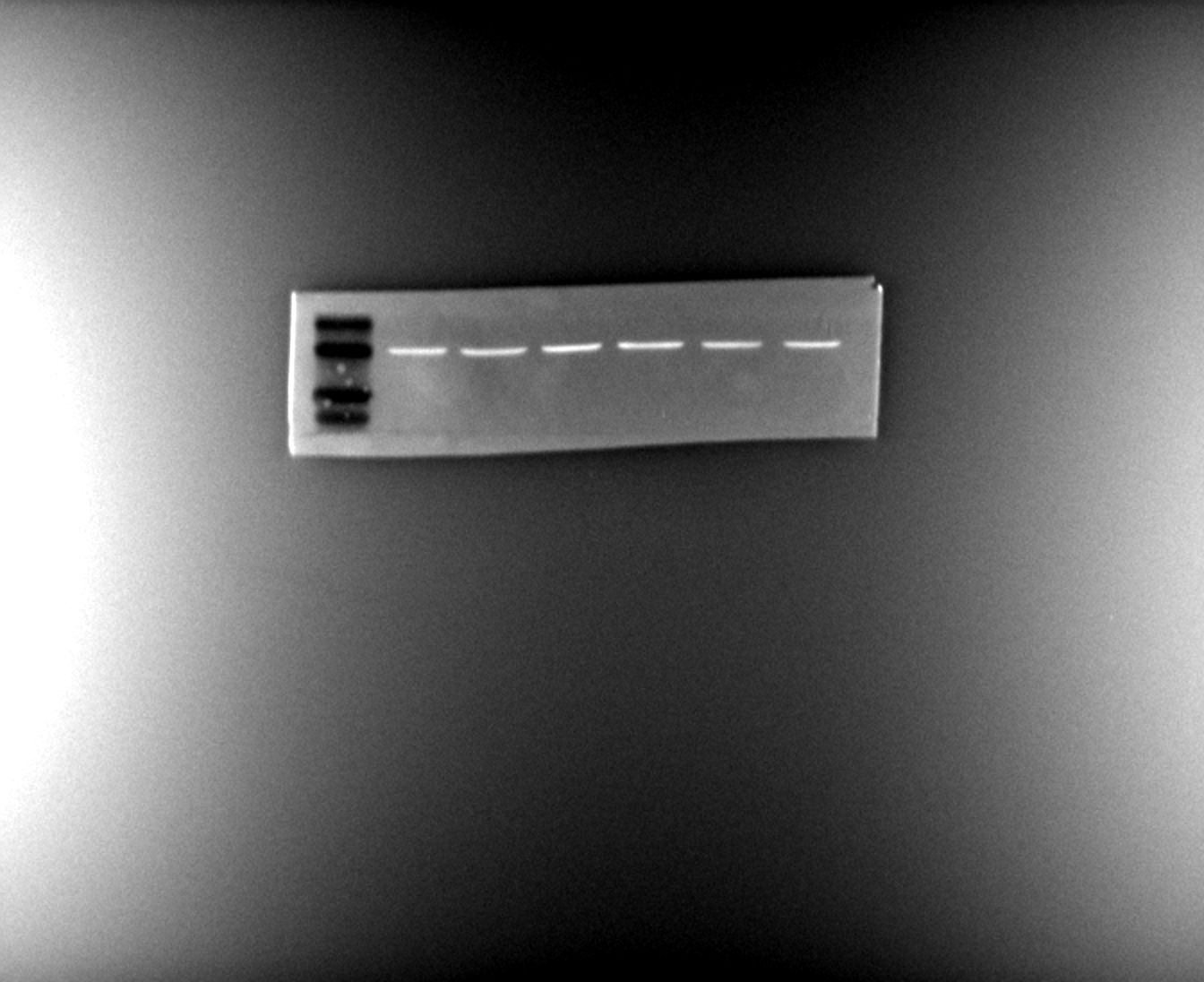

Supplement: Supplementary file 2 [file Data_Sheet_1.ZIP › RAW data MS ID1763548/Figure 2/Figure 2F WB images/a┬-actin/a┬-actin 3 PVDF membrane.tif]

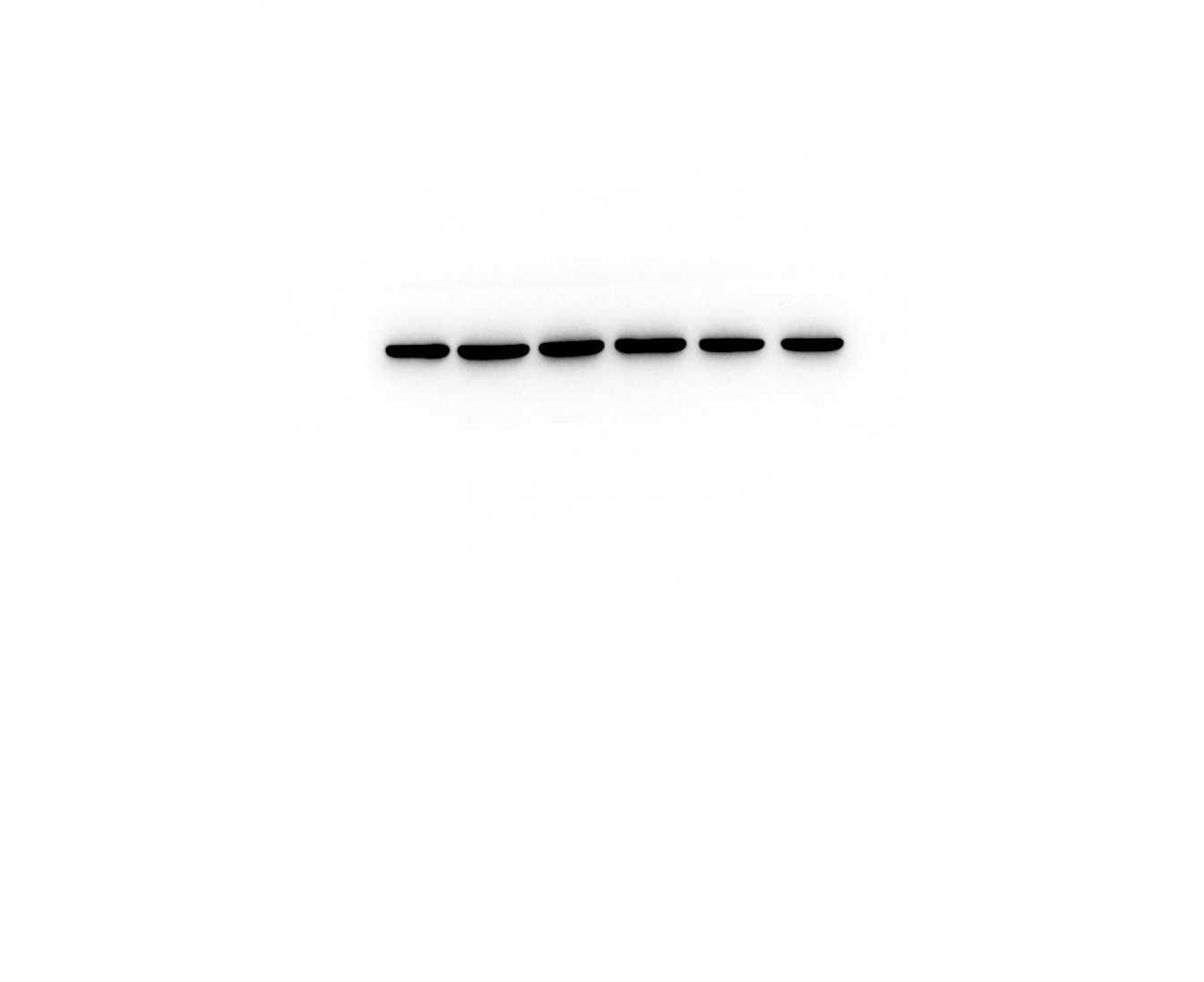

Supplement: Supplementary file 2 [file Data_Sheet_1.ZIP › RAW data MS ID1763548/Figure 2/Figure 2F WB images/a┬-actin/a┬-actin 3.tif]
